# Feature fusion context attention gate UNet for detection of polycystic ovary syndrome

**DOI:** 10.1038/s41598-025-26314-4

**Published:** 2025-11-27

**Authors:** Yuvaraj Natarajan, Sri Preethaa K. R., Shyamala Devi M.

**Affiliations:** 1 School of Computer Science and Engineering, Vellore Institute of Technology, Vellore, Tamil Nadu 632014 India; 2 Department of Robot and Smart System Engineering, Kyungpook National University, 80, Daehak-ro, Buk-gu, Daegu, 41566 Republic of Korea

**Keywords:** Polycystic ovary syndrome (PCOS), DL, Feature fusion context attention U-Net (FCAU-Net), Ultrasound imaging, Medical image classification, Computational biology and bioinformatics, Diseases, Endocrinology, Health care, Mathematics and computing, Medical research

## Abstract

Polycystic Ovary Syndrome (PCOS) is a prevalent endocrine disorder affecting women of reproductive age, characterized by hormonal imbalance, irregular menstrual cycles, and ovarian cysts. Traditional diagnostic approaches, which include clinical evaluations, radiological studies, and surgical interventions, are often time-consuming, costly, and not always reliable. To improve the accuracy and efficiency of PCOS diagnosis, this research introduces the Feature Fusion Context Attention U-Net (FCAU-Net) model, leveraging deep learning (DL) techniques. This study makes two key contributions. First, it enhances dataset preparation through Fuzzy Contrast Enhanced (FCE) imaging. Second, it integrates a Feature Fusion Context (FFC) module into the Attention U-Net model, optimizing the extraction of context and position weights from feature maps for better classification performance. An openly available PCOS Ultrasound Image Dataset with 3,800 images was partitioned with 80: 20 to ensure that only original images were used for testing, while augmented samples were exclusively utilized for training to enhance model generalization and robustness. The remaining 3040 images was augmented to form 45,600 images and split into training and validation sets in an 80:20 ratio. The augmented images were processed and tested with several DL models, including DenseNet, AlexNet, VGG19, ResNet, U-Net, and Attention U-Net. Among these, the Attention U-Net initially achieved over 80% accuracy in detecting PCOS. The proposed FCAU-Net, which incorporates the FFC module, demonstrated superior performance, achieving a detection accuracy of 99.89%, significantly outperforming existing DL models. This research highlights the potential of FCAU-Net in providing a more accurate and efficient tool for the diagnosis of PCOS.

## Introduction

Polycystic Ovary Syndrome (PCOS) is a major medical condition with a high prevalence of morbidity and mortality across the world. Proper care and prevention of related problems including diabetes, heart disease, and infertility depend on early identification of PCOS. Since PCOS appears differently in each person, developing a consistent set of diagnostic criteria is difficult. PCOS diagnostic requirements can cause inconsistent diagnosis amongst medical professionals and different regions, which can affect the precision of treatment plans and occurrence estimations. Medical practitioners may find it difficult to diagnose PCOS because of a number of factors including a patient’s history, complex hormonal profiles, overlap with other conditions, subjective ultrasound interpretation, diagnostic delay, and varied symptoms. A typical technique for visualizing ovarian morphology in PCOS patients is transvaginal ultrasonography. Interpreting ultrasonography results, such as ovarian volume and cyst presence, can be subjective and operator-specific. The accuracy and consistency of the PCOS diagnosis may be compromised by this variation. DL has the potential to improve diagnostic consistency and accuracy by providing a consistent, objective method of evaluating large, complicated information, such as clinical data and medical images. In recent years, there has been a fast development of DL techniques, which have been employed in medical image analysis and disease classification. DL has demonstrated outstanding performance in a number of medical specialties, including skin diseases, pathological conditions, and radiography. DL models may use massive data sets and complex algorithms to uncover hidden patterns and characteristics from medical imaging. The adaptation of DL methods may result in faster and more accurate diagnosis times. The neural networks are used in SL that extract knowledge from massive volumes of data. Complex characteristics may be automatically extracted from medical images using DL models, which may help with the objective interpretation of ultrasound scans used to diagnose PCOS. In terms of PCOS, DL offers promising opportunities to improve and automate PCOS screening. Automatic PCOS identification speeds up diagnosis and enhances medical practitioners’ diagnostic consistency. Medical professionals may minimize disparities between radiologists’ diagnoses by employing DL technology to automate and standardize the PCOS detection process. This consistency increases the standard of care given to patients, reduces the incidence of diagnostic mistakes, and increases the precision of diagnoses.

CNNs that focus on segmentation, like AResUNet, CR-UNet, and Ocys-Net, have been investigated in recent studies on PCOS detection using ultrasound imaging. These CNNs mainly improve follicle and cyst localization, but they frequently have difficulties with generalized feature representation. Although deep CNN-based classifiers such as VGG16, EfficientNetB6, and SqueezeNet have demonstrated remarkable classification accuracy, their ability to adapt to noisy clinical ultrasound pictures is limited due to their heavy reliance on transfer learning and large-scale data. Although they offer better diagnostic robustness, hybrid models that combine CNNs with ensemble learners (such as SVM, RF, or BiLSTM) or feature selection methods have a greater computational cost. Elman NN and PCOS-WaveConvNet, two wavelet and spectral-based models, have improved spatial-spectral extraction but lack precise contextual awareness. While attention-based designs such as ASPPNet and Attention U-Net have demonstrated superior multiscale feature learning, they frequently lack in their ability to successfully incorporate position-sensitive information and contextual dependencies. With the above challenges, this research proposes FCAU-Net model that offers the PCOS detection with high accuracy. To address these limitations, the proposed FCAU-Net introduce an innovative FFC module that adaptively combines spatial, positional, and contextual cues from feature maps, while FCE preprocessing enhances cystic boundary visibility.

### Paper organization and research contribution

This research paper is organized as follows: Sect. 2 explores the background literature review. Section 3 deals with the design of proposed FCAU-Net model research methodology. Section 4 covers the mathematical modeling of the proposed FCAU-Net model. Section 5 discusses the results and implementation analysis of the proposed FCAU-Net model. In the end, Sect. 6 concludes the proposed FCAU-Net model that includes the challenges, novelty and Future work. This research primarily contributes in two ways.

**Fig. 1:**
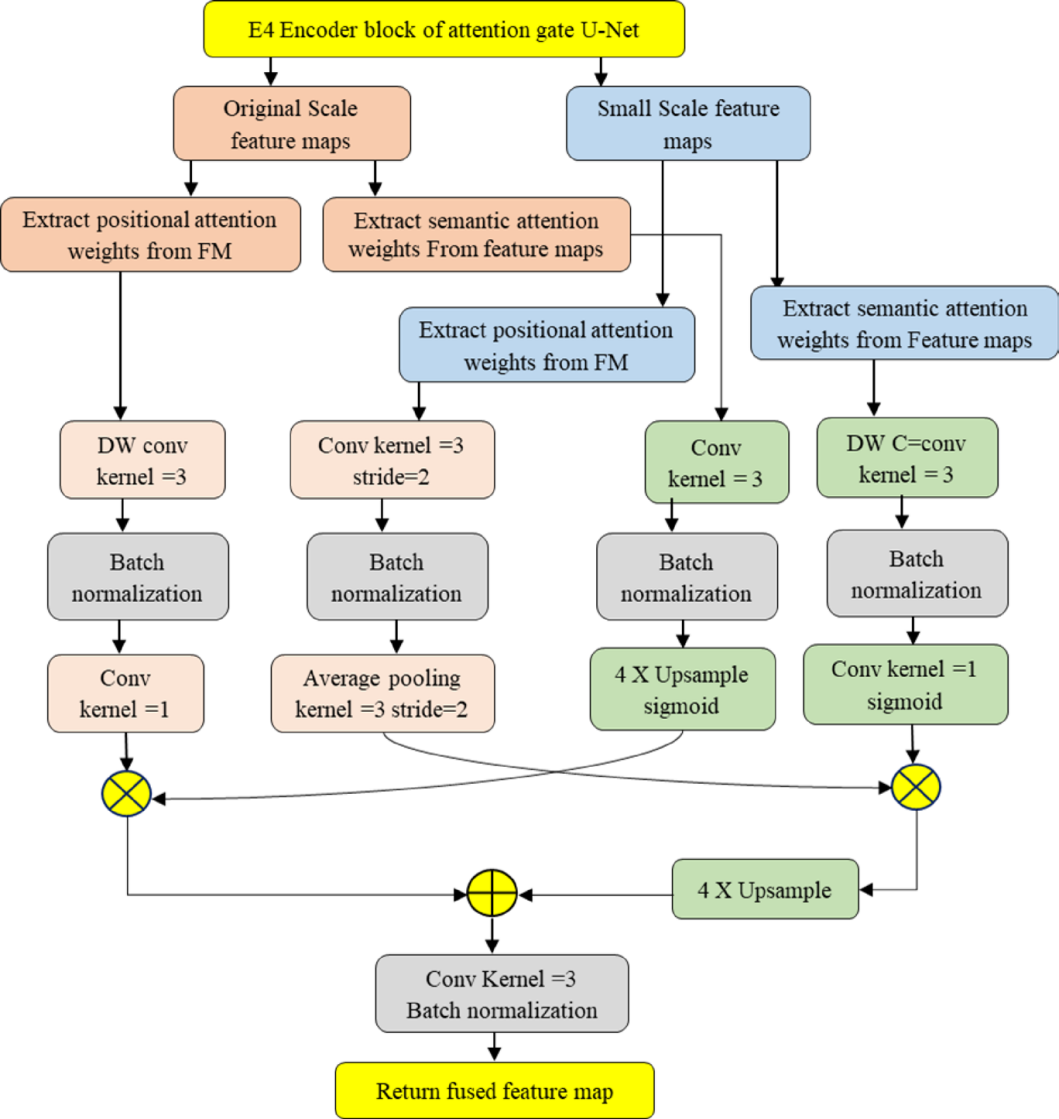
Feature fusion context module.

(i) *Fuzzy Contrast Enhancement*: The first contribution focuses on preparing datasets through image cropping and feature improvement using an adaption of the FCE image. FCE is the novel preprocessing technique that improves the quality of ultrasound images by enhancing contrast and reducing ambiguity, thereby facilitating more accurate feature extraction.
(ii) *Feature Fusion Context Attention U-Net*: The second contribution emphasizes on integrating FFC module into the Attention U-Net model for supervised learning towards detecting PCOS. Proposes enhanced Attention U-Net architecture FCAU-Net that is integrated with a FFC module that effectively captures both contextual and positional information from feature maps, leading to superior diagnostic performance. The FFC module extracts the Context and position weights of the Feature Maps (FM) that optimizes the deep and shallow features of FM as shown in Fig. 1.

## Background study

Several DL and machine learning approaches have been explored for the detection and classification of PCOS using medical imaging, particularly ultrasound. Early studies applied CNN-based architectures with segmentation and handcrafted preprocessing techniques, such as adaptive bilateral filtering, Otsu thresholding, watershed segmentation, and Gabor wavelet-based feature extraction, to enhance cyst and follicle identification. Models such as AResUNet and CR-UNet demonstrated improved noise reduction, robustness against low-contrast images, and better adaptation to multimodal inputs. The following Table 1 presents a structured overview of existing works on PCOS detection and related medical image analysis, highlighting the methodology, imaging modality, preprocessing techniques, and performance outcomes.

**Table 1:** Key outcomes from the related works.

Model	Preprocessing	Approach	Key outcome
*Segmentation-focused CNN:* AResUNet^1^, CR-UNet^22^, Ocys-Net^17^, CNN with Segmentation^9^, Threshold and Watershed^21,39^, Hybrid^37^, GrabCut^41^	Filtering, Otsu, contour, watershed	Deep U-Net variants, reverse bottleneck, watershed and contour-based segmentation	Improved follicle, cyst localization and tracking
*Deep CNN classifiers:* VGG16^10^, SqueezeNet^26^, CNN + FC^27^, ITL-CNN^19^, InceptionV3^7,28^, EfficientNetB6^38^	Ultrasound preprocessing, TL, normalization	CNN-based classification with batch norm, TL, activation optimization	Higher PCOS detection and classification accuracy
*Hybrid DL models:* VGG16 + Ensemble^8,20^, CNN with BiLSTM^12^, CNN with KNN^15^, CNN with SVM, DT, RF^29,40^, Sequential 2D CNN with FS^31^	Feature selection, ensemble integration	CNN fused with ensembles, clustering, sequential learning, wrapper FS	Robust diagnosis across diverse datasets
*Wavelet and spectral approaches:* PCOS-WaveConvNet^6^, Elman NN with Wavelet^32^, BPA with Gabor^35^	Spectral domain, Gabor wavelets	Wavelet-based ConvNets, Elman NNs, Gabor filters	Strong spectral, spatial feature extraction
*GAN and augmentation methods:* GAN with CNN^4^	Data augmentation	Generative adversarial networks with CNN classifier	Overcame overfitting, improved generalization
*Feature-driven and hybrid Methods* (ESDPCOS^13^, AMCNN^14^, MLOD^16^, GIST-MDR^42^, Probabilistic PCOS^33^	Ultrasound texture, GLCM, fuzzy logic	Hybrid models combining CNN with GLCM, fuzzy logic, texture scoring	Enhanced robustness in classification
*Attention and context learning* (ASPPNet + ResNet^11^, AMCNN^14^, EfficientNetB6 with Attention UNet^38^	Ultrasound	Attention mechanisms, dilated conv, spatial pyramid pooling	Better multiscale learning and context capture
*Other imaging modalities:* Ovarian quantification^2^, Eye scleral biomarkers^3^, MRI follicle count^36^	Ultrasound, scleral, MRI	Quantification of follicles, scleral biomarkers, MRI follicle mapping	Multimodal biomarkers for improved PCOS detection

To address the limitations of conventional CNNs, attention mechanisms and multi-scale learning architectures were introduced. Examples include ASPPNet, AMCNN, and ensemble frameworks combining EfficientNet and Attention U-Net, which enabled better contextual feature extraction and improved segmentation efficiency. Similarly, transfer learning strategies with InceptionV3, VGG16, and ResNet variants allowed reuse of pre-trained weights for enhanced classification performance on limited datasets. Hybrid approaches also emerged to overcome challenges of feature uncertainty and overfitting. These integrated deep models with traditional techniques such as fuzzy logic, SVMs, clustering, or wavelets. For instance, CNNs combined with fuzzy layers or KNN clustering improved feature reliability, while GAN-based augmentation addressed overfitting by generating synthetic data. Other models, such as PCOS-WaveConvNet and Ocys-Net, explored wavelet transforms and reverse bottleneck designs for richer feature representation. Recent advancements focused on ensemble and optimization-driven methods, including stacking models that merged VGG16, ResNet50, and MobileNet, or HHO-DQN frameworks that optimized hyperparameters for deep networks. Additionally, segmentation-driven workflows, such as hybrid Otsu-Chanvese segmentation, GrabCut with fuzzy SNN models, and probabilistic grid-based analysis, provided more precise localization of follicles. Overall, the existing works achieved varying levels of accuracy, but faced persistent challenges on handcrafted preprocessing, and limited robustness across datasets. This underscores the need for an end-to-end, adaptive, and context-aware architecture, which motivates the development of proposed FCAU-Net. The DL applications towards health care with pre-trained CNN models can be explored^43–46^. The inferences, advantages and the limitations from literature survey were shown in Table 2.

**Table 2:** Inferences from literature Review.

Methodology and Inference		Advantages	Limitations
Sequential CNN	Fuzzy CNN^5^, Confluence CNN^12^, ESDPCOS^13^, AMCNN^14^, KNN based CNN^15^, MLOD^16^, Ocys-Net^17^, ITL-CNN^19^, Ensemble CNN^20^, Hybrid CNN^23^, Sequential CNN^27^, 2D CNN^29^, Tri-stage wrapper CNN^31^, SSFSE-DL^36^, DLNNSVM^40^, GrabCut and Fuzzy Logic -SNN Model^41^	Best in handling images with permissible noise and scalable to apply the same model to any large number of datasets Easy to integrate with other CNN architectures or combined with TL Image pixels and the ROI are identified uniquely in each convolution layer operation	The model should be refined with labelled data Need to validate with various cross-validation methods to fine-tune the accuracy Need separate validation to handle augmentation images Depends on feature filtering to extract deep features from the ultrasound images
Recurrent CNN	Complex Spatial Recurrent Neural Network U-Net^22^, Elman NN^32^, Back Propagation Algorithm^35^	Spatial relationship between image pixels is effectively correlated Relationship between the image frames is reused Effectively extract the patterns in ultrasound image	Best suited for large dataset Less performance while handling image with ling sequences and cannot handle static image data Depends on effective feature extraction methods
Pre-Trained CNN	AResUNet^1^, Inception CNN^2^, ResNet^4^, VGGNet^4^, Inception V3^4,28^, PCOS-WaveConvNet^6^, PCONet^7^, VGG16^8,9,24,30^, ASPPNet^10^, HHO-DQN^18^, SqueezeNet^26^, UNet and EfficientNet^38^, GIST-MDR^42^, U-Net^3^ and ResNet^3^	AResUNet achieves 98% of accuracy Inception CNN achieves 84.81% of accuracy Exhibits good performance with little amount of data Best in extracting the deep features	Model need to be fine-tuned with several activation function and number of layers to improve the performance Depends on balanced data before fitting the model

Pathological image segmentation method^58^ based on multiscale and dual attention mechanisms, aiming to enhance feature representation and improve segmentation precision. The multiscale module allows the network to capture both global contextual information and fine-grained local details, while the dual attention mechanism emphasizes salient spatial and channel-wise features, reducing the influence of irrelevant regions. Improved TransUnet framework^59^ for melanoma image segmentation on integrating transformer-based global context modeling with enhanced convolutional modules could capture fine-grained lesion details with high accuracy. The high-order paired-ASPP (Atrous Spatial Pyramid Pooling) Network^60^ enhances the semantic segmentation by effectively capturing both global context and fine local structures. By leveraging high-order feature interactions and a paired atrous spatial pyramid pooling design, the method improves boundary delineation and reduces semantic ambiguity. EnsembleEdgeFusion framework^61^ designed to advance semantic segmentation in microvascular decompression imaging. By integrating multiple segmentation models with edge-aware fusion strategies, the method enhances boundary precision and structural consistency in complex medical images. Dilated SE-DenseNet^62^ framework classifies the brain tumor using MRI scans. By combining dilated convolutions with squeeze-and-excitation modules, the model captures multi-scale contextual information while adaptively emphasizing the most relevant features.

The automated framework for high-precision PCOS detection that leverages the Segment Anything Model (SAM)^63^ applied to super-resolution ultrasound ovary images. By combining advanced segmentation with image enhancement, the method achieves more accurate follicle boundary delineation and improved feature representation. HR-ASPP^64^, an enhanced semantic segmentation model for cervical nucleus images builds on DeepLabv3 + with improved atrous spatial pyramid pooling. By focusing on high-resolution spatial localization and robust shape feature extraction, the model achieves more precise nucleus boundary detection. The dual-stage U-Net DSU-Net^65^ integrates CNN-based local feature extraction with transformer-based global context modeling for skin lesion segmentation. This hybrid design enhances both boundary precision and contextual understanding, enabling more accurate lesion delineation. The dual-encoder attention network DEAU-Net^66^ improve medical image segmentation by combining two encoders with attention mechanisms. It effectively captures multi-scale contextual information and emphasizes salient features while suppressing irrelevant regions. The integration of attention mechanisms with DL enhances the medical image segmentation by feature representation, capturing contextual dependencies, and improving segmentation accuracy across diverse imaging modalities. These approaches have shown remarkable performance in diverse medical imaging domains, including brain tumor classification, melanoma segmentation, cervical nucleus detection, and microvascular imaging, highlighting the potential of attention-guided and hybrid feature extraction frameworks. Inspired by these research works, the proposed FCAU-Net integrates feature-calibrated attention modules with an end-to-end architecture tailored for ovarian ultrasound images, addressing challenges such as small follicle structures, low contrast, and imaging noise. The combination of multiscale feature extraction, attention-guided focus, and computational efficiency in FCAU-Net is directly motivated by these prior works, aiming to achieve high-precision, robust, and clinically applicable PCOS detection, surpassing the limitations of existing CNN, U-Net, and ensemble-based approaches.

Recent advancements in medical image processing have witnessed the emergence of several U-Net derivatives that integrate hybrid architectural components such as Atrous Spatial Pyramid Pooling (ASPP), dual attention schemes, and Squeeze-and-Excitation (SE) modules to enhance feature extraction and boundary precision. For instance, SAP-UNet has been successfully employed for ultrasound-based segmentation by combining ASPP with SE blocks to capture multi-scale contextual representations while refining channel-wise significance^72^. Similarly, DDA-AttResUNet^73^ developed for breast and ovarian ultrasound segmentation tasks, utilizes a dual decoder mechanism fused with residual and attention pathways to enhance feature propagation between encoder and decoder stages. Beyond these, architectures such as DA-TransUNet^74^ and Hybrid Dilated Residual U-Net^75^ have incorporated both spatial and channel-wise attention blocks for fine-grained tissue segmentation. In the specific context of ovarian and PCOS imaging, studies such as CystNet, Enhanced AResU-Net^76^, Follicles-Net^77^, RNN^78^ and ML^79^ have reported improved cyst recognition through hierarchical or dilated convolutional blocks. These designs, while effective in multiscale feature aggregation, often focus on static pooling or global context enhancement without fully integrating localized spatial relational learning. The proposed FCAU-Net differs by introducing FFC module that dynamically combines spatial and contextual information across scales, thereby refining ovarian cyst segmentation boundaries and follicular classification accuracy. Moreover, through FCE preprocessing, the model ensures improved noise resilience and region smoothness compared to ASPP or SE-based variants.

## Research methodology of proposed FCAU-Net model

**Fig. 2:**
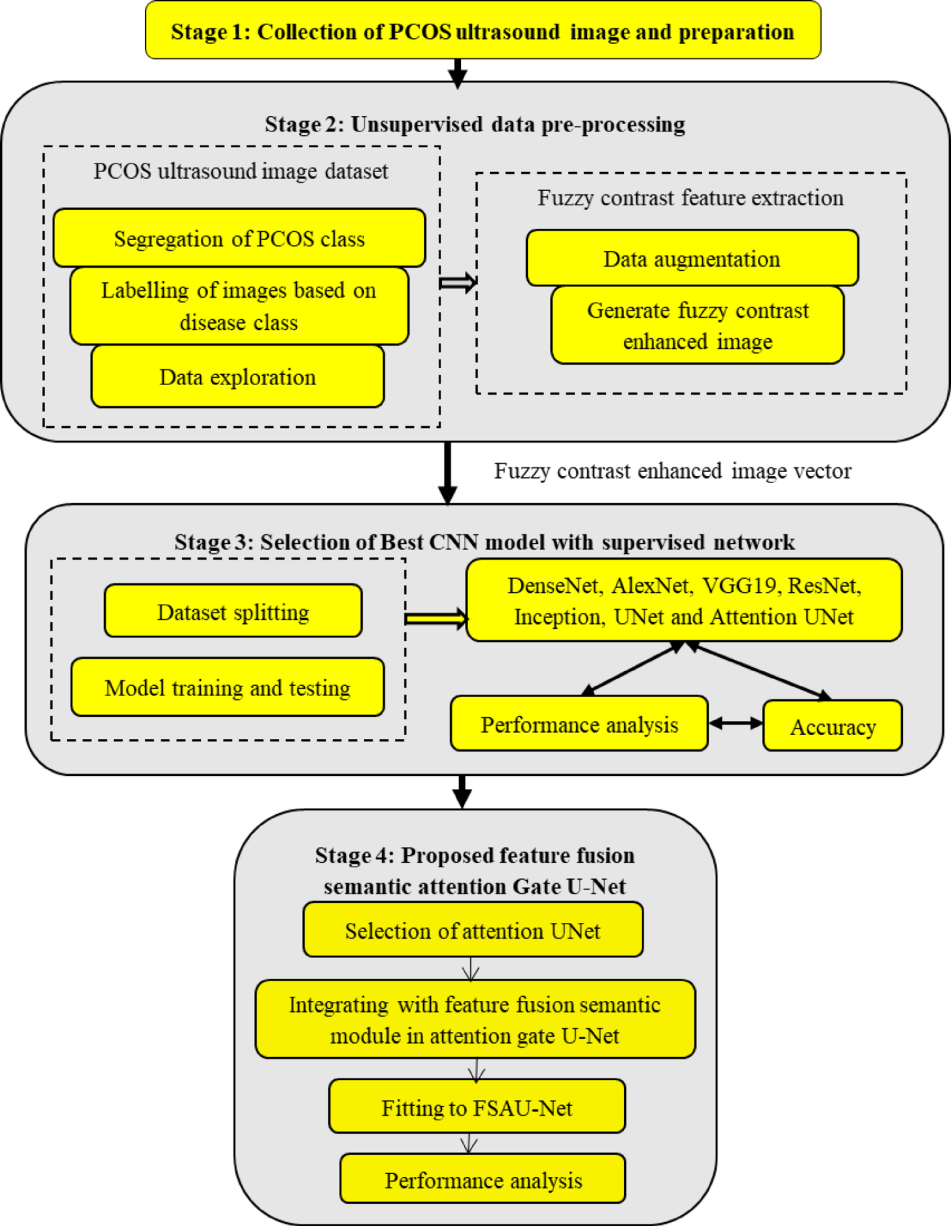
Proposed FCAU-Net research methodology.

The proposed FCAU-Net model was designed to classify the PCOS infected and Normal healthy images. The FCAU-Net research methodology is shown in Fig. 2. The overall research methodology of FCAU-Net initiates with stage 1 that performs collection of 3800 PCOS ultrasound Images Dataset from KAGGLE having 1900 PCOS infected images and 1900 Normal healthy images^47^. Stage 2 deals with dataset preprocessing that segregates the images based on normal and PCOS symptoms. Then the Labelling of the image is done followed by data augmentation by generating 14 augmented images for each image in the dataset resulting with 53,200 images. The data augmentation was performed using horizontal flipping, vertical flipping, rotation with positive and negative angle of 45, 90, 135, 180, 225, 270 degrees. The data augmented cropped images are subjected to generate fuzzy contrast enhanced image vector. Stage 3 fits the HEG images are fitted with the existing CNN models like DenseNet, AlexNet, VGG19, ResNet, Inception, UNet and Attention UNet to select the best CNN model. The Attention U-Net found to detect the existence of PCOS with accuracy above 80%.

**Fig. 3:**
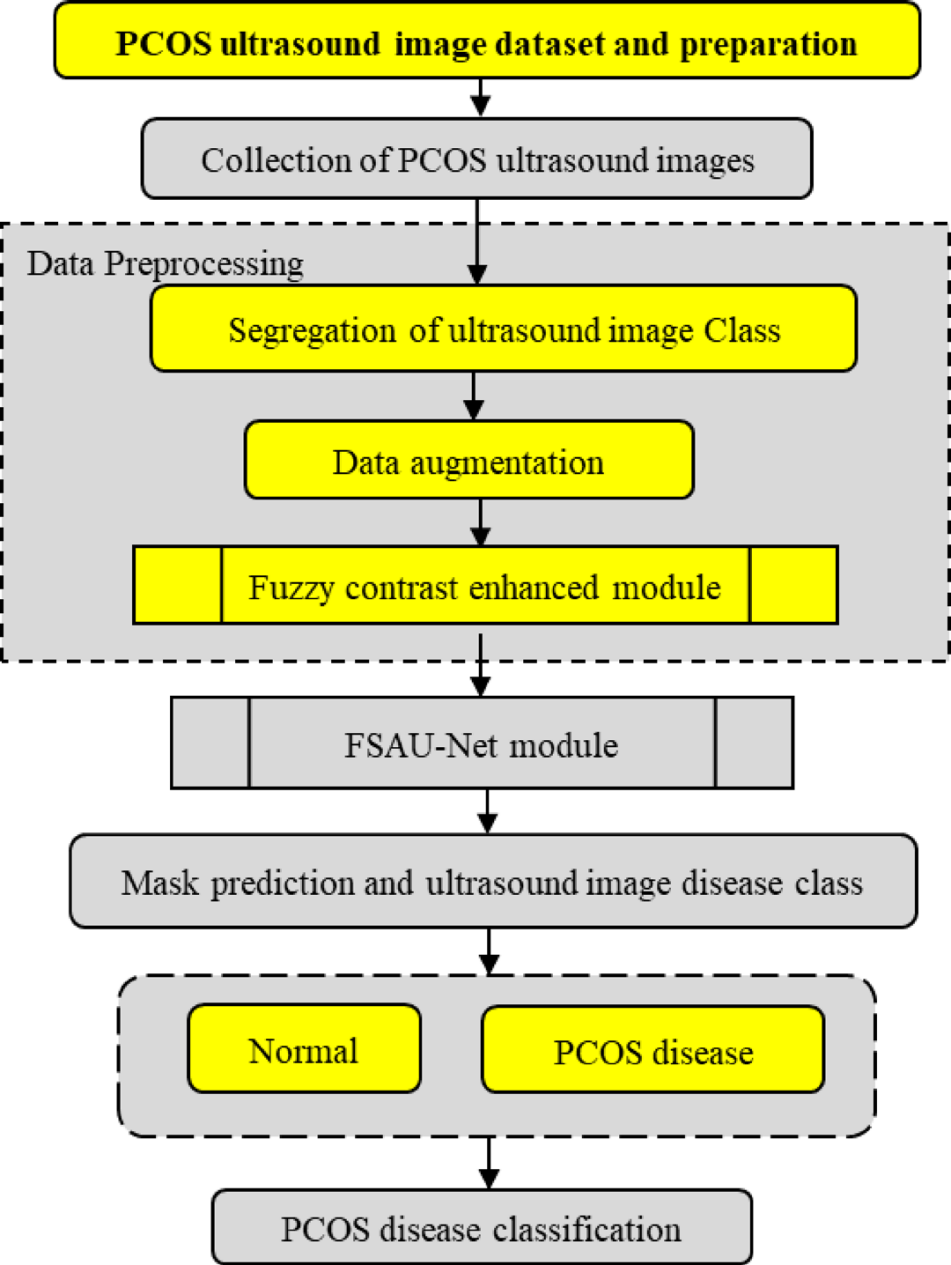
Overall architecture of proposed FCAU-Net.

**Fig. 4:**
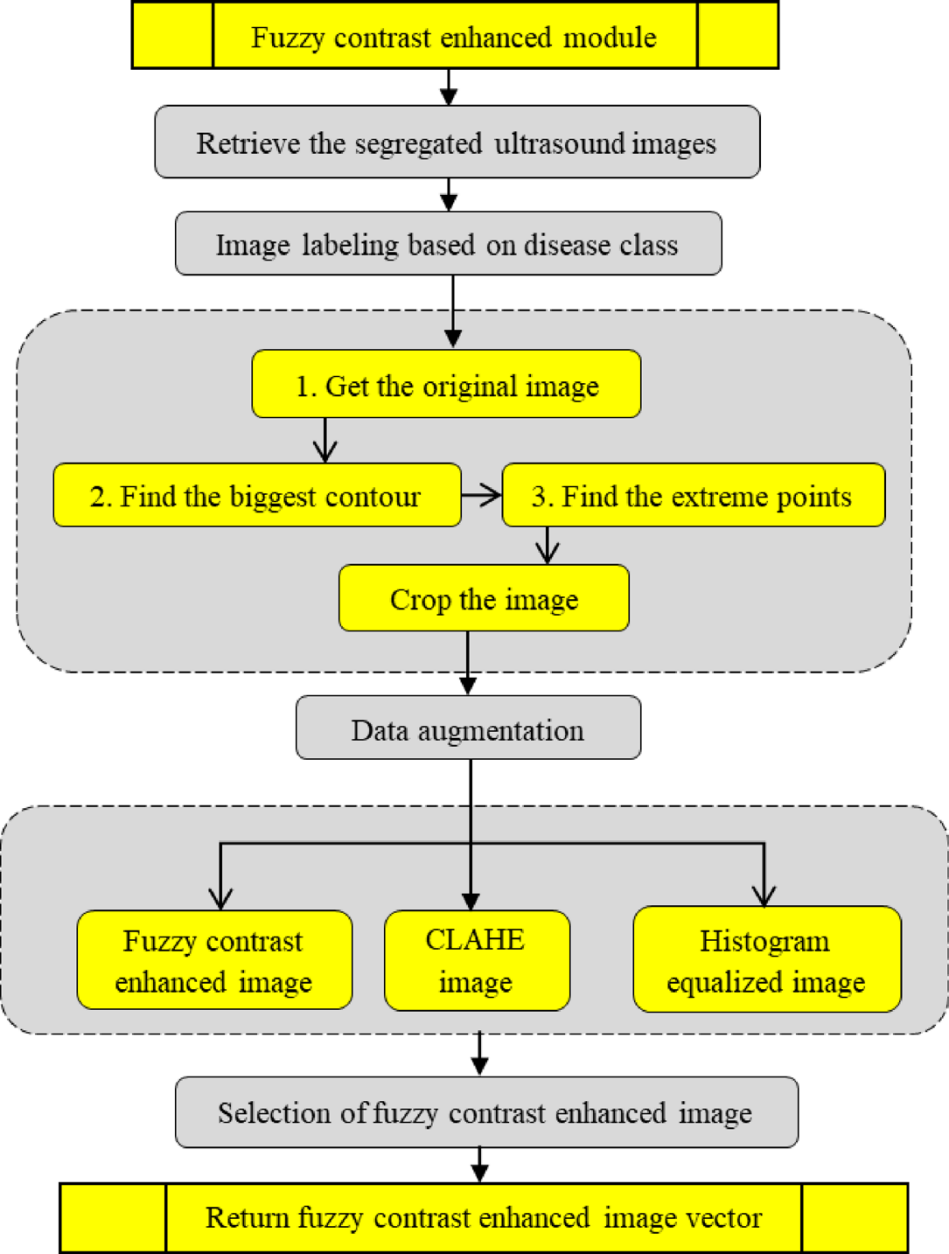
Steps in generating enhanced image vector.

So, the Attention U-Net was refined to improve the accuracy by proposing FCAU-Net. The proposed FCAU-Net Overall architecture shown in Fig. 3. The FCAU-Net framework retrieves the PCOS ultrasound images that are subjected to the image segregation based on the disease class as Normal and PCOS infected images. The PCOS ultrasound images are performed with data preprocessing to generate FCE images by calling Fuzzy contrast enhanced module that is shown in Fig. 4. The FCE images are fitted to proposed FCAU-Net framework that predicts the mask and classifies the ultrasound images based on the class. Steps involved in generating the enhanced image vector (Fig. 4) retrieves the segregated labeled PCOS ultrasound images.

**Fig. 5:**
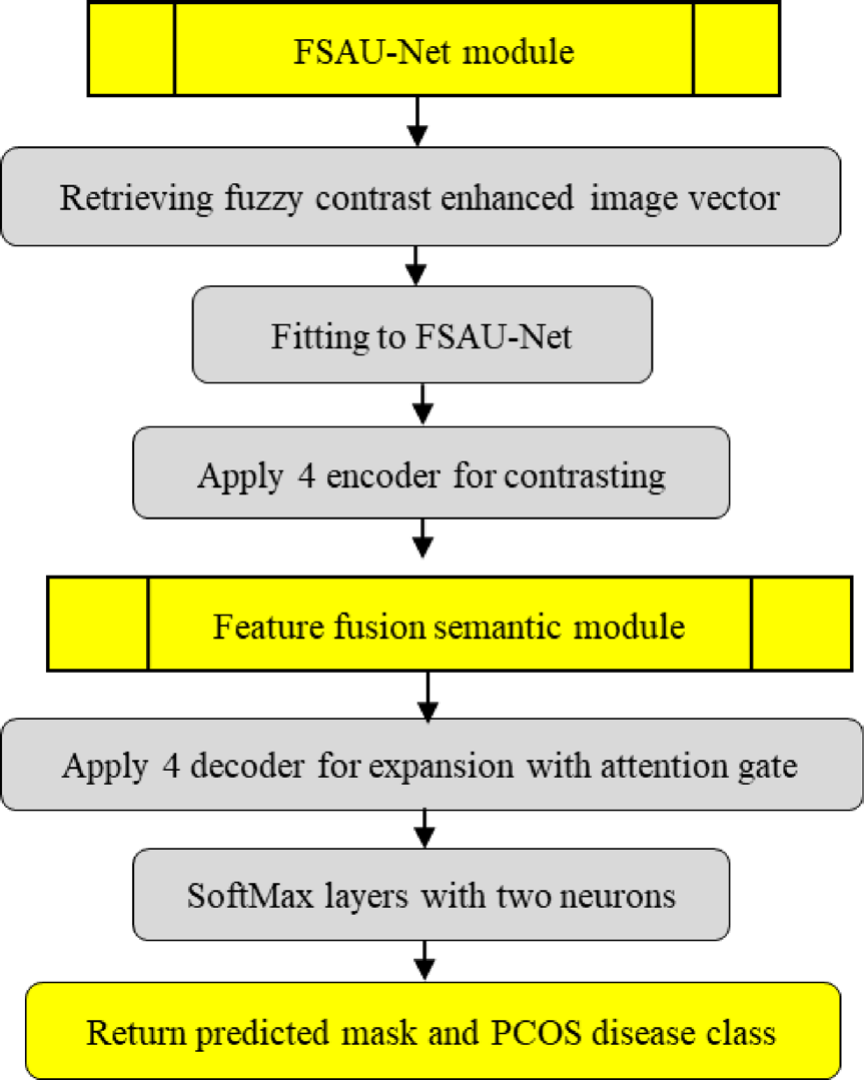
FCAU-Net framework.

The labelled PCOS ultrasound images subjected to image cropping by finding the biggest contour and extract the extreme points to form the crop images. The image cropping was performed in order to focus on the significant image features. The cropped images are performed with data augmentation that 14 images for each image in the dataset resulting with 53,200 images. All the data augmented images are subjected to enhance the brightness of the image by forming Histogram equalized image, CLAHE image and Fuzzy Contrast Enhanced image. This work forms both Histogram equalized image and CLAHE with the intent of performance evaluation as both of them serves a distinct purpose. The normal Histogram equalization of the image works for the entire image by stretching the pixel intensity. Though CLAHE is a method of histogram equalization, it applies histogram for small adaptive regions that prevents noise in homogenous areas. The FCE images, known for their high brightness, are validated using the PSNR ratio and processed through the Fuzzy Contrast Enhanced module. The FCAU-Net framework, depicted in Fig. 5, uses these FCE images as input. The images are passed through four encoder blocks and four decoder blocks. The encoder-decoder feature maps are combined using the Feature Fusion Context (FFC) module, which extracts positional and contextual characteristics to generate optimized fused feature maps.

**Fig. 6:**
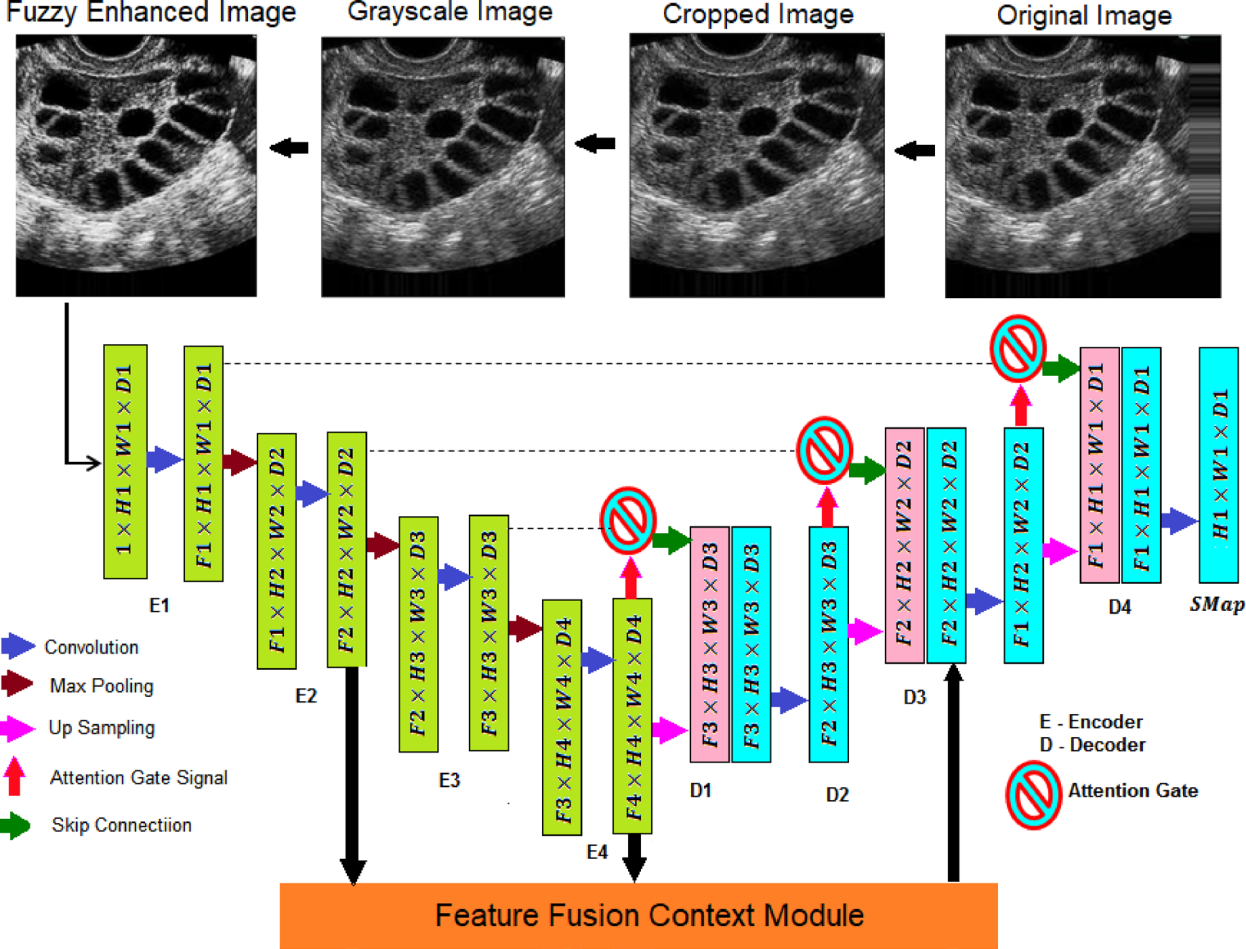
FCAU-Net Framework (F – Feature Maps, H, W, D – Height, width and Depth of the feature maps).

In the FCAU-Net (Fig. 6), the FCE images are initially downscaled by a factor of 2 through the encoder blocks, creating contrasted FCE feature maps. These are then passed through decoder blocks integrated with attention gates, which upscale the feature maps, resulting in expanded FCE segmentation feature maps.

**Fig. 7:**
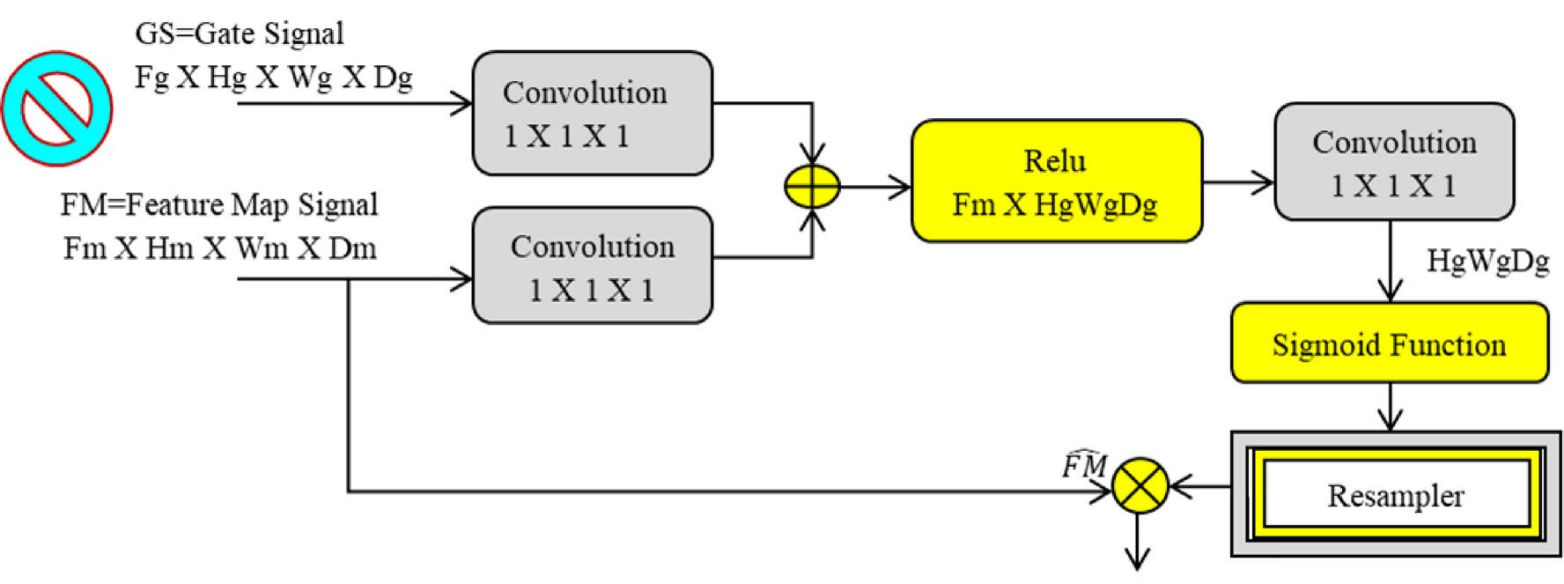
Attention gate network in FCAU-Net.

The attention gate mechanism is illustrated in Fig. 7. The attention gate in FCAU-Net takes the input feature map and gate signal to calculate the gating coefficient. Batch normalization is applied to the gating coefficient to center the features in the active region while maintaining the relevance of unaligned weights in the feature maps. ReLU activation is then used to introduce nonlinearity, helping the feature vector learn complex representations. Dropout is applied to remove noise from the aligned weights of the feature map. A 1 × 1 linear convolution is performed next to generate the attention feature map based on vector concatenation. Finally, the sigmoid activation function is applied, assigning a weight of “1” to the aligned features to create the attention coefficient feature map.

### Feature fusion context module in proposed FCAU-Net

The novelty of this research lies in the integration of the Feature Fusion Context (FFC) Module, positioned between the encoder and decoder blocks of the FCAU-Net, as shown in Fig. 1. The FFC module enhances feature maps by extracting positional and contextual information. Positional information is obtained by analysing correlations within feature maps (FMs). Spatial features are extracted through convolution, resulting in a 3D FM comprising query, key, and value components. These features are compared to compute the Energy Score Matrix (ESM), which highlights the relative importance of pixel positions. The ESM is normalized using SoftMax to generate Position Attention Weights (PAW), capturing positional details. Contextual information is derived by identifying interdependencies between FM channels. Attention scores indicating feature importance are computed and normalized with SoftMax to create weighted FMs. These weighted FMs are multiplied with the original FMs to strengthen the cumulative channel content. The weighted and original FMs are then integrated to enhance the model’s ability to capture contextual details. The FFC module processes both original and small-scale FMs to fuse positional and contextual information, resulting in improved feature map quality.

This fusion significantly enhances prediction performance, validating the module’s effectiveness in optimizing feature. First, the Position Attention Weights (PAW) are generated for both original and small-scale feature maps (FMs). For the original scale FM, depth-wise convolution with three kernels is applied, followed by batch normalization and single-kernel convolution. For the small-scale FM, convolution with three kernels and two strides is followed by batch normalization and average pooling with three kernels and two strides. Next, the Semantic Attention Weights (SAW) are created. For the original scale FM, convolution with three kernels is applied, followed by batch normalization and up-sampling with a sigmoid activation function. For the small-scale FM, depth-wise convolution with three kernels is followed by batch normalization and single convolution with a sigmoid activation function. The PAW and SAW of the original scale FM are then combined to form the Position Semantic Weight for the original scale FM. Similarly, the PAW and SAW of the small-scale FM are concatenated and up-sampled to create the Position Semantic Weight for the small-scale FM. Finally, the Position Semantic Weights of both scales are fused to produce optimized fused FM.

## Development of FCAU-Net and mathematical modeling

The FCAU-Net initiates by collecting ultrasound images from publicly available PCOS ultrasound Images Dataset from KAGGLE dataset for classifying the PCOS infected and normal healthy images are denoted in the Eq. (1).

1 $$PCOS_{{3800}} = \left\lfloor { \cup _{{W = 1}}^{{3800}} \left\{ {\sum\nolimits_{{e = 1}}^{{255}} {\sum\nolimits_{{d = 1}}^{{255}} {PCOS_{{ed}} } } } \right\}} \right\rfloor$$

Where “${{PCOS}_{00}}_{1}$” denotes single ultrasound image, “$e,\:d$” denotes the number of row and column pixels and “$W$” denotes the number of images in the dataset. The single ultrasound image is denoted in Eq. (2).

2 $$PCOS_{{001}} = \left[ {\begin{array}{*{20}l} {PCOS\left( {0,0} \right)} & {PCOS\left( {0,1} \right)} & { \ldots \ldots \ldots } & {PCOS\left( {0,255} \right)} \\ {PCOS\left( {1,0} \right)} & {PCOS\left( {1,1} \right)} & { \ldots \ldots \ldots } & {PCOS\left( {1,255} \right)} \\ \vdots & \vdots & { \ldots \ldots \ldots } & \vdots \\ {PCOS\left( {255,0} \right)} & {PCOS\left( {255,1} \right)} & { \ldots \ldots \ldots } & {\:PCOS\left( {255,255} \right)} \\ \end{array} } \right]$$

The ultrasound images are applied to the data preprocessing module by processing fuzzy contrast enhancement to generate the FCE images.

### Data preprocessing modeling

The input ultrasound images are segregated based on the PCOS disease class as shown in the Eq. (3) denoting 1900 “$\:Infect$” as PCOS infected images and 1900 “$\:Norm$” as normal healthy images which were labeled.

3 $$PCOS_{{3800}} = \left\lfloor { \cup _{{W = 1}}^{{1900}} \left\{ {\sum\nolimits_{{e = 1}}^{{255}} {\sum\nolimits_{{d = 1}}^{{255}} {Infect_{{ed}} } } } \right\} + \cup _{{W = 1}}^{{1900}} \left\{ {\sum\nolimits_{{e = 1}}^{{255}} {\sum\nolimits_{{d = 1}}^{{255}} {Norm_{{ed}} } } } \right\}} \right\rfloor$$

The labeled ultrasound images are processed with the image cropping and data augmentation.

### Image cropping and data augmentation modeling

Each labeled “${{PCOS}_{00}}_{1}$” ultrasound input image is processed with image cropping by extracting the biggest contour and extract the ultrasound image extreme points to form the cropped images “${{CropImgPCOS}_{00}}_{1}$” as shown in Eq. (4) to Eq. (5)

4 $$\:Contour=maxcontour\left({{PCOS}_{00}}_{1}\right)$$

5 $$\:{{CropImgPCOS}_{00}}_{1}=Extremepoints\left(Contour\right)\:\:$$

The cropped images “${{CropImgPCOS}_{00}}_{1}$” are processed with data augmentation to form PCOS augmented images “${{AugPCOS}_{00}}_{1}$” resulting with 53,200 images. The data augmentation was performed using horizontal flipping from the Eqs. (6) to (7). Here, “$\:CropImgPCOS$” denotes cropped image patch showing the horizontally cropped ROI portion from the main PCOS dataset image, “$Horizontal\:Flip$” denotes the horizontal transformation matrix that performs horizontal mirroring. The variables “$e,\:d$” denotes the coordinate variables representing the column and row pixel positions respectively in the image that define the location of each pixel before horizontal flipping transformation. The “$[c;e;1]$” denotes original homogeneous coordinate vector having the column vector form of the pixel coordinate before horizontal flipping. The “$[c{\prime\:};e{\prime\:};1]$” denotes the transformed coordinate vector representing the pixel coordinates after horizontal flipping.

6 $$\:\left[{{CropImgPCOS}_{00}}_{1}\right]={\left[\begin{array}{c}c\\\:\begin{array}{c}e\\\:1\end{array}\end{array}\right]=Horizontal\:Flip\left[{{CropImgPCOS}_{00}}_{1}\right]}^{{\prime\:}}=\left[\begin{array}{c}c\:{\prime\:}\\\:\begin{array}{c}e\:{\prime\:}\\\:1\end{array}\end{array}\right]$$

7 $$Horizontal\;Flip = \left[ {\begin{array}{*{20}c} {c^{\prime } } \\ {\begin{array}{*{20}c} {e^{\prime } } \\ 1 \\ \end{array} } \\ \end{array} } \right] = \left[ {\begin{array}{*{20}c} { - 1} \\ {\begin{array}{*{20}c} {\begin{array}{*{20}c} 0 \\ \vdots \\ \end{array} } \\ 0 \\ \end{array} } \\ \end{array} \begin{array}{*{20}c} 0 \\ {\begin{array}{*{20}c} {\begin{array}{*{20}c} 1 \\ \vdots \\ \end{array} } \\ 0 \\ \end{array} } \\ \end{array} \begin{array}{*{20}c} \cdots \\ {\begin{array}{*{20}c} {\begin{array}{*{20}c} \ldots \\ \ldots \\ \end{array} } \\ \ldots \\ \end{array} } \\ \end{array} \begin{array}{*{20}c} 0 \\ {\begin{array}{*{20}c} {\begin{array}{*{20}c} 0 \\ \vdots \\ \end{array} } \\ 1 \\ \end{array} } \\ \end{array} } \right] \times \left[ {\begin{array}{*{20}c} c \\ {\begin{array}{*{20}c} e \\ 1 \\ \end{array} } \\ \end{array} } \right]$$

The vertical flipping operation is shown from Eqs. (8) to 9). Here, “$\:CropImgPCOS$” denotes cropped image patch showing the vertical cropped ROI portion from the main PCOS dataset image, “$Vertical\:Flip$” denotes the vertical transformation matrix that performs vertical mirroring. The variables “$f,\:g$” denotes the coordinate variables representing the column and row pixel positions respectively in the image that define the location of each pixel before vertical flipping transformation. The “$[f;g;1]$” denotes original homogeneous coordinate vector having the column vector form of the pixel coordinate before vertical flipping. The “$[f{\prime\:};g{\prime\:};1]$” denotes the transformed coordinate vector representing the pixel coordinates after vertical flipping.

8 $$\:\left[{{CropImgPCOS}_{00}}_{1}\right]={\left[\begin{array}{c}f\\\:\begin{array}{c}g\\\:1\end{array}\end{array}\right]=Vertical\:Flip\left[{{CropImgPCOS}_{00}}_{1}\right]}^{{\prime\:}}=\left[\begin{array}{c}f\:{\prime\:}\\\:\begin{array}{c}g\:{\prime\:}\\\:1\end{array}\end{array}\right]$$

9 $$\:Vertical\;Flip = \left[ {\begin{array}{*{20}c} {f^{\prime } } \\ {\begin{array}{*{20}c} {g^{\prime } } \\ 1 \\ \end{array} } \\ \end{array} } \right] = \left[ {\begin{array}{*{20}c} 1 \\ {\begin{array}{*{20}c} {\begin{array}{*{20}c} 0 \\ \vdots \\ \end{array} } \\ 0 \\ \end{array} } \\ \end{array} \begin{array}{*{20}c} 0 \\ {\begin{array}{*{20}c} {\begin{array}{*{20}c} { - 1} \\ \vdots \\ \end{array} } \\ 0 \\ \end{array} } \\ \end{array} \begin{array}{*{20}c} \cdots \\ {\begin{array}{*{20}c} {\begin{array}{*{20}c} \ldots \\ \ldots \\ \end{array} } \\ \ldots \\ \end{array} } \\ \end{array} \begin{array}{*{20}c} 0 \\ {\begin{array}{*{20}c} {\begin{array}{*{20}c} 0 \\ \vdots \\ \end{array} } \\ 1 \\ \end{array} } \\ \end{array} } \right] \times \left[ {\begin{array}{*{20}c} f \\ {\begin{array}{*{20}c} g \\ 1 \\ \end{array} } \\ \end{array} } \right]$$

The rotation operation is shown in the equation Eq. (10) to (11). Here, “$\:CropImgPCOS$” denotes cropped image patch showing the rotated portion from the main PCOS dataset image, “$\:Rotation$” denotes the rotation transformation matrix that applies geometric rotation in homogeneous space. The variables “$m,\:n$” denotes the coordinate variables representing the column and row pixel positions respectively in the image that define the location of each pixel before rotation transformation. The “$[m;n;1]$” denotes original homogeneous coordinate vector of the pixel coordinate before rotation. The “$[m{\prime\:};n{\prime\:};1]$” denotes the transformed coordinate vector representing the pixel coordinates after rotation.

10 $$\:\left[{{CropImgPCOS}_{00}}_{1}\right]={\left[\begin{array}{c}m\\\:\begin{array}{c}n\\\:1\end{array}\end{array}\right]=Rotation\left[{{CropImgPCOS}_{00}}_{1}\right]}^{{\prime\:}}=\left[\begin{array}{c}m\:{\prime\:}\\\:\begin{array}{c}n\:{\prime\:}\\\:1\end{array}\end{array}\right]$$

11 $$Rotation = \left[ {\begin{array}{*{20}c} {m^{\prime } } \\ {\begin{array}{*{20}c} {n^{\prime } } \\ 1 \\ \end{array} } \\ \end{array} } \right] = \left[ {\begin{array}{*{20}c} {\cos \theta } \\ {\begin{array}{*{20}c} {\begin{array}{*{20}c} { - \sin \theta } \\ \vdots \\ \end{array} } \\ 0 \\ \end{array} } \\ \end{array} \begin{array}{*{20}c} {\sin \theta } \\ {\begin{array}{*{20}c} {\begin{array}{*{20}c} {\cos \theta } \\ \vdots \\ \end{array} } \\ 0 \\ \end{array} } \\ \end{array} \begin{array}{*{20}c} \cdots \\ {\begin{array}{*{20}c} {\begin{array}{*{20}c} \ldots \\ \ldots \\ \end{array} } \\ \ldots \\ \end{array} } \\ \end{array} \begin{array}{*{20}c} 0 \\ {\begin{array}{*{20}c} {\begin{array}{*{20}c} 0 \\ \vdots \\ \end{array} } \\ 1 \\ \end{array} } \\ \end{array} } \right] \times \left[ {\begin{array}{*{20}c} m \\ {\begin{array}{*{20}c} n \\ 1 \\ \end{array} } \\ \end{array} } \right]$$

The final resultant data augmentation results "$AugPCOS$ " are obtained as depicted in Eqs. (12) to (15), with “$\:HFPCOS$” denoting the horizontally flipped image, denoting “$\:VFPCOS$” the vertically flipped image and “$\:RPCOS$” denoting the rotated image.

12 $$\:HFPCOS=Horizontal\:Flip\left(CropImgPCOS\right)\:$$

13 $$\:VFPCOS=Vertical\:Flip\left(CropImgPCOS\right)\:$$

14 $$\:RPCOS=Rotation\:\left(CropImgPCOS\right)\:$$

15 $$\:AugPCOS=\bigcup\:\left\{\begin{array}{c}HFPCOS\\\:VFPCOS\\\:RPCOS\end{array}\right.$$

The data augmented images are processed with fuzzy contrast enhanced module.

### Fuzzy contrast enhanced module modeling

The data augmented ultrasound images “$\:AugPCOS$” are processed to form the Histogram equalized images, CLAHE image and FCE images. The histogram equalized ultrasound image “${{HistPCOS}_{00}}_{1}$” was formed by applying cumulative distribution function “$\:cdf\:$” of each image pixel and histogram variance “$\:Histogram$” as in Eqs. (16) to (17) denoting “$(x,y)$” are the pixel co-ordinates of data augmented ultrasound image. Here $\:\text{min}\left(cdf\right)$ denotes the smallest cumulative probability value used for normalization. The “$Width\left(dots\right)$” denotes the number of pixels in the horizontal dimension of the image. The “$Height\left(dots\right)$” denotes the number of pixels in the vertical dimension of the image. The “$RC,\:GC,\:BC$” denotes the red, green and blue channel intensity component of the augmented image.

16 $$\:Histogram=round\:\left(\frac{cdf\left({{AugPCOS}_{00}}_{1}\left(x,y\right)-\text{min}\left(cdf\right)\right)}{Width\left(dots\right)\:\times\:Height\left(dots\right)-\text{m}\text{i}\text{n}\left(cdf\right)}\times\:\left(RC+GC+BC\right)-1\right)$$

17 $$\:{{HistPCOS}_{00}}_{1}={{AugPCOS}_{00}}_{1}+Histogram$$

As the data augmented ultrasound images “$\:AugPCOS$” is an array of pixel values which are denoted by the array of numbers as random dots. Suppose if “$(x,y)$” are the two random pixel variables and if they are exactly linearly correlated with constant “$c$”, then it is shown in Eq. (18).

18 $$\:{{AugPCOS}_{00}}_{1}\left(x\right)=c{\:\times\:{AugPCOS}_{00}}_{1}\left(y\right)$$

Now the probability density function (PDF) of the two random dots is denoted as Eq. (19) where ‘r’ is the total number of roots of (31) which is equal to ‘1’. Here $\:\frac{d\left({y}_{j}\right)}{d\left(x\right)}$ represents how intensity values “${y}_{j}$” change with respect to “$x$” during transformation.

19 $$PDF\left( {AugPCOS_{{001}} \left( x \right)} \right) = \sum\nolimits_{{j = 1}}^{r} f \left( {AugPCOS_{{001}} (y_{j} )} \right)\left| {\frac{{d\left( {y_{j} } \right)}}{{d\left( x \right)}}} \right|$$

In CLAHE, the PDF inside the local region of the augmented ultrasound image was found and is denoted by Eq. (20), where “${PDF}_{LR}\left({{AugPCOS}_{00}}_{1}\left(x\right)\right)$” represents the contrast of the augmented ultrasound image by CLAHE and is substituted to “$Q$” as in Eq. (21).

20 $$\:{PDF}_{LR}\left({{AugPCOS}_{00}}_{1}\left(x\right)\right)\ne\:1$$

21 $$\:{PDF}_{LR}\left({{AugPCOS}_{00}}_{1}\left(x\right)\right)=Q$$

The value of $\:"Q"$ lies between 0 and 1. If $\:"Q=1"$, then the local histogram stretching is maximum, but in CLAHE the value of $\:"Q"$ must be less than ‘1’, since the contrast stretching is limited. The value of $\:"Q"$ is denoted in Eq. (22)

22 $$\:{Q=\:PDF}_{LR}\left({{AugPCOS}_{00}}_{1}\left(y\right)\right)\left|\frac{d\left(y\right)}{d\left(x\right)}\right|LR$$

Where$\:{\:"PDF}_{LR}\left({{AugPCOS}_{00}}_{1}\left(y\right)\right)"$ is the PDF of the local region “$\:LR\:$” of original augmented ultrasound image and “$\:LR$”, $\:\left|\frac{d\left(y\right)}{d\left(x\right)}\right|$ is the ratio $\:d\left(y\right),\:d\left(y\right)$ of the image in that local region. By integrating on both sides of Eq. (22), we get the form as in Eq. (23). The transformation function of image contrast with CLAHE is given in Eq. (24) denoting $\:"k"$ as integral constant. The contrast enhanced image by CLAHE $\:{{"CLAHEPCOS}_{00}}_{1}"\:$is given in Eq. (25)

23 $$\:Q*{\int\:}_{LR}^{x}dx=\:{\int\:}_{LR}^{x}{PDF}_{LR}\left({{AugPCOS}_{00}}_{1}\left(y\right)\right)\:dy$$

24 $$\:{{{AugPCOS}_{00}}_{1}\left(x\right)}_{LR}\:\:=\frac{1}{Q}*{\int\:}_{LR}^{x}{PDF}_{LR}\left({{AugPCOS}_{00}}_{1}\left(y\right)\right)\:dy+\:k$$

25 $$\:{{CLAHEPCOS}_{00}}_{1}={{{AugPCOS}_{00}}_{1}\left(x\right)}_{LR}\:\:$$

As the input augmented ultrasound images “$\:AugPCOS\:$” is in the gray scale format of size $\:"M\:\times\:\:N"$ with “$\:GL\:$” gray levels with $\:"gl=0,\:\text{1,2}\dots\:.L-1"$. The gray levels were defined as the group of fuzzy sets representing the membership pixel value to the image property as in Eq. (26) for the single augmented ultrasound image $\:"{{AugPCOS}_{00}}_{1}"$. The notation of the fuzzy sets $\:"\frac{{\mu\:}_{mn}}{{gl}_{mn}}"$ represents the fuzzy membership of the $\:"M\:\times\:\:N"$ pixel.

26 $$AugPCOS_{{001}} = \cup _{{m = 1}}^{M} \cup _{{m = 1}}^{N} \frac{{\mu _{{mn}} }}{{gl_{{mn}} }}\:\:where\:\mu \:_{{mn}} \: \in \:[0,\:1]$$

The fuzzy contrast image $\:"FCE\left(Y\right)"$ of the input augmented ultrasound images “$\:AugPCOS$” was formed by performing three processes as fuzzification $\:"{\Phi\:}"\:$operation, Membership value $\:"{\Gamma\:}"$ operation, and defuzzification$\:\:"{\Psi\:}"$ operation as denoted by Eq. (27)

27 $$\:FCE\left(Y\right)={\Psi\:}\left(\:{\Gamma\:}\left(\:{\Phi\:}\left({{AugPCOS}_{00}}_{1}\:\right)\right)\right)$$

The modified gray levels$\:{\:\:"GL}^{{\prime\:}}"$ of the augmented ultrasound image $\:"{{AugPCOS}_{00}}_{1}"$ is computed as in Eq. (28).

28 $$GL^{\prime } = f\left( {AugPCOS_{{001}} } \right) = (GL - 1)\sum\nolimits_{{x = 0}}^{{gl}} {\frac{{h\left( i \right)}}{{MN}}}$$

The gray level fuzzification $\:"{\Phi\:}"\:$operation is performed on the image $\:"{\mu\:}_{mn}"\:$membership value as in Eq. (29) denoting as exponential “$\:FE$” and denominational “$\:FD$” fuzzifiers that control the amount of grayness level in the FCE image.

29 $$\:{\mu\:}_{mn}\left(gl\right)={\left[1+\frac{{gl}_{max}-\:gl}{FD}\:\right]}^{-FE}$$

Now, the Membership value $\:"{\Gamma\:}\left(gl\right)"$ operation on the image is performed as shown in Eq. (30)

30 $$\:{\Gamma\:}\left(gl\right)=\left\{\begin{array}{l}{2\left[{\mu\:}_{mn}\left(gl\right)\right]}^{2}\\\:1-{2\left[{\mu\:}_{mn}\left(gl\right)\right]}^{2}\end{array}\right.\begin{array}{c}\:\:\:\:\:\:\:if\:0\le\:{\mu\:}_{mn}\left(gl\right)\le\:0.5\:\\\:\:\:\:\:\:if\:0.5<{\mu\:}_{mn}\left(gl\right)\le\:1\end{array}$$

The defuzzification$\:\:"{\Psi\:}"$ operation on the image is performed as shown in Eq. (31) and the FCE image was found from Eq. (32) and Eq. (33).

31 $$\:{\Psi\:}\left(gl\right)=\left\{\begin{array}{c}n-\left(n-{gl}_{\text{m}\text{i}\text{n}}\right)(1-2{\mu\:}_{mn}\left(gl\right))\\\:n+\left({gl}_{\text{m}\text{a}\text{x}}-n\right)(2{\mu\:}_{mn}\left(gl\right)-1)\end{array}\right.\begin{array}{c}\:\:\:\:\:\:\:if\:0\le\:{\mu\:}_{mn}\left(gl\right)\le\:0.5\:\\\:\:\:\:\:\:if\:0.5<{\mu\:}_{mn}\left(gl\right)\le\:1\end{array}$$

32 $$\:{{FCEPCOS}_{00}}_{1}\left(x,y\right)=\:FCE\left({{AugPCOS}_{00}}_{1}\right)$$

33 $$\:FCE\left({{AugPCOS}_{00}}_{1}\right)={\Psi\:}\left(\:{\Gamma\:}\left(\:{\Phi\:}\left({{{{\mu\:}_{mn}(AugPCOS}_{00}}_{1})}_{gl}\:\right)\right)\right)\:$$

The brightness and Pixel Intensity of the obtained Histogram equalized images, CLAHE image and FCE images are validated to select high pixel intensity image. The pixel intensity $\:"Int(x,y)"\:$was validated by finding the scene transmission $\:"t(x,y)"\:$distant dependent factor of the images. The scene radiance $\:"SR(x,\lambda\:)"\:$was also estimated denoting$\:"\lambda\:"\:$as transmission coefficient. The validation of pixel intensity for histogram equalized image $\:"IntHist\left(x,y\right)"\:$was done as in Eq. (34) to Eq. (35) with$\:"\beta\:"$ representing as color density, $\:\:"d"$ distant dependent constant factor and$\:{\:"L}_{\alpha\:}\left(\lambda\:\right)"$ as scattering coefficient.

34 $$\:t\left({{HistPCOS}_{00}}_{1}\left(x,y\right)\right)=\:{e}^{-\beta\:\:.\:\:d}$$

35 $$\:IntHist\left(x,y\right)=SR\left(x,\lambda\:\right)+{L}_{\alpha\:}\left(\lambda\:\right)\:\left[1-t\left({{HistPCOS}_{00}}_{1}\left(x,y\right)\right)\right]$$

The validation of pixel intensity for CLAHE image $\:"IntCLAHE\left(x,y\right)"\:$was done as in Eq. (36) to Eq. (37) with$\:\:"\beta\:"$ representing as color density, $\:\:"d"$ distant dependent constant factor and$\:{\:"L}_{\alpha\:}\left(\lambda\:\right)"$ as scattering coefficient.

36 $$\:t\left({{CLAHEPCOS}_{00}}_{1}\left(x,y\right)\right)=\:{e}^{-\beta\:\:.\:\:d}$$

37 $$\:IntCLAHE\left(x,y\right)=SR\left(x,\lambda\:\right)+{L}_{\alpha\:}\left(\lambda\:\right)\:\left[1-t\left({{CLAHEPCOS}_{00}}_{1}\left(x,y\right)\right)\right]$$

The validation of pixel intensity for FCE image$\:\:"IntFCE\left(x,y\right)"$ was done in Eq. (38) to Eq. (39)

38 $$\:t\left({{FCEPCOS}_{00}}_{1}\left(x,y\right)\right)=\:{e}^{-\beta\:\:.\:\:d}$$

39 $$\:IntFCE\left(x,y\right)=SR\left(x,\lambda\:\right)+{L}_{\alpha\:}\left(\lambda\:\right)\:\left[1-t\left({{HighGAS}_{00}}_{1}\left(x,y\right)\right)\right]$$

The high pixel intensity image was selected based on comparing the obtained pixel intensity of Histogram equalized images, CLAHE image and FCE images as in Eq. (40). The pixel intensity of FCE was found to be high. The FCE images are applied to FCAU-Net module

40 $$\:HighPixel=max\left\{\begin{array}{l}IntHist\left(x,y\right)\\\:IntCLAHE\left(x,y\right)\\\:IntFCE\left(x,y\right)\end{array}\right.$$

### Proposed FCAU-Net PCOS detection modeling

The fuzzy contrast enhanced image $\:"{{FCEPCOS}_{00}}_{1}"\:$input data is processed with existing CNN models to select the best CNN model. Experiment results portray that Attention UNet offers the classification of PCOS with the accuracy above 80%. Now the Attention UNet was selected to integrate the feature fusion context module. The FCAU-Net consists of four encoder block and four decoder blocks accompanied with the Attention gate. The feature map produced by the convolution after each encoder is shown in Eq. (41) that defines the feature map $\:{FEMap}_{mn}\:$at position $\:"(m,n)"$, which results from applying the convolution operator $\:"Con\left(i,j\right)"\:$between the image pixels “$\:PIXx$” and the convolution kernel “$\:KER$”.

41 $$\:{FEMap}_{mn}=Con\left(i,j\right)={\left(PIXx*KER\right)}_{ij}$$

The convolution expansion operation is shown in Eq. (42) with $\:"{PIX}_{i-r,j-c}"\:$denoting the pixel intensity from the input image at position $\:\left(i-r,j-c\right)$ in the receptive field with $\:(i,\:j)$ as pixel coordinates. Here $\:{"KER}_{r,c}"\:$denotes the kernel coefficient that is the weight at position $\:(r,c)$ in the convolution kernel.

42 $$\left( {PIX*KER} \right)_{{ij}} = \sum\nolimits_{{r = 1}}^{{255}} {\sum\nolimits_{{c = 1}}^{{255}} {PIX_{{i - r,j - c}} *KER_{{r,c}} } }$$

After the convolution, the sigmoid function “$\:Sig$” was performed as in Eq. (43) where $\:"\sigma\:\left(.\right)"$ Is the Sigmoid function. Here $\:{"FEM}_{mn}"\:$denotes the feature map intensity which is the raw convolution output before activation. The $\:{"e}^{-{FEM}_{mn}}"$ denotes the exponential decay term that controls how steeply the sigmoid transitions between 0 and 1.

43 $$\:Sig=\sigma\:\left({FEM}_{mn}\right)\frac{1}{1+\:{e}^{-{FEM}_{mn}}}$$

### Feature fusion context module modeling

The FFC module was integrated between the encoder and decoder block of the FCAU-Net. The FFC module extracts the position and Context information of the FM. The Context information of the FM was computed by finding the interdependencies between different channels and extracts attention scores that indicate the feature importance. The attention scores are normalized with SoftMax to form the attention weights distributions with probabilistic values to form Weighted FM. Assume “$\:FEMap$” is the obtained feature map which is shown in Eq. (44) with $\:"C\:\times\:\:H\:\times\:W"$ denoting the channel, height and width of FM. The context attention scores “$\:AS$” of the feature map were formed in the format of channel attention map “$\:AM$” as in Eq. (45).

44 $$\:\:AM={FEMap}^{C\:\times\:\:H\:\times\:W}$$

45 $$\:{AS}_{ij}=\frac{\text{exp}\left({AM}_{i}{AM}_{j}\right)}{{\sum\:}_{i=1}^{c}\text{exp}\left({AM}_{i}{AM}_{j}\right)}$$

The attention scores $\:{"AS}_{ij}"\:$are multiplied with the original FM to form the cumulative FM channel content. Now, the weighted FM was integrated with the original FM to regulate the FM strength. This validates that the model can learn the context details “$\:CD$” of the FM with the attention distribution of the FM pixels with scale parameter $\:"\beta\:"$ as in Eq. (46).

46 $$\:CD=\beta\:{\sum\:}_{i=1}^{c}\text{exp}\left({AS}_{ij}{AM}_{i}\right)+{AM}_{j}$$

The position information of the FM was done by analysing the correlation between the position in the FM. The process starts by acquiring the spatial features from FM by applying convolution that results in 3D FM consisting of query, key, and value. By comparing the 3D FM features, the Energy Score Matrix (ESM) was computed. Assume “$\:FEMap$” is obtained FM. The Position Attention Weights “$\:PW$” of the FM were formed in format of “$\:EM$” as in Eq. (47) and Eq. (48)

47 $$\:\:EM={FEMap}^{C\:\times\:\:H\:\times\:W}$$

48 $$\:{PW}_{ij}=\frac{\text{exp}\left({EM}_{i}{EM}_{j}\right)}{{\sum\:}_{i=1}^{c}\text{exp}\left({EM}_{i}{EM}_{j}\right)}$$

The ESM represents the relative importance between FM pixel positions. The ESM are then normalized with softmax to form Position Attention Weights “$\:PW$” that shows the position information of FM. This validates that model can learn the position details “$\:PD$” of the FM with the Position Attention Weights “$\:PW$” of the FM pixels with scale parameter $\:"\beta\:"$ as in Eq. (49).

49 $$\:PD=\beta\:{\sum\:}_{i=1}^{c}\text{exp}\left({PW}_{ij}{EM}_{i}\right)+{EM}_{j}$$

The FFC module extracts the position and Context information of both the original and small-scale feature maps. First, the PAW of the original and small-scale feature maps is formed. The PAW of the original scale FM is processed with depth wise convolution using 3 kernels followed by batch normalization and single kernel convolution to form PAW of original scale FM. The PAW of original scale FM is denoted by $\:"{FMO}_{Pos}"$ that denotes the “position block 1”. The SAW of the small-scale FM is processed with depth wise convolution using 3 kernels followed by batch normalization and single convolution with sigmoid activation function to form SAW of small-scale FM. The SAW of small-scale FM is denoted by $\:"{FMS}_{Con}"$ that denotes the “context block 1”. The $\:"{FMO}_{Pos}"$and$\:\:"{FMS}_{Con}"\:$is denoted as in Eq. (50) and Eq. (51). The operation performed on SAW of small-scale FM as in Eq. (52) with $\:"\text{FM}"$ denoting FM.

50 $$\:{FMO}_{Pos}\in\:{FM}^{H\times\:W\times\:C}$$

51 $$\:{FMS}_{Con}\in\:{FM}^{\frac{H}{4}\times\:\frac{W}{4}\times\:C}$$

52 $$FM_{{CV}}^{C} [m,n] = \sum\nolimits_{{i = 1}}^{M} {\sum\nolimits_{{j = 1}}^{N} {DK_{{ij}}^{C} FMS_{{Con}}^{C} [m + i,n + j]} }$$

The value $\:"{FM}_{CV}^{C}[m,n]"$ represents the context value of the $\:"{C}^{th}"$ channel at position $\:"[m,n]"$. The $\:{DK}_{ij}^{C}$ denotes the depth wise kernel of the $\:"{C}^{th}"$ channel at position $\:"[i,j]"$. The $\:"{FMS}_{Con}^{C}\left[m+i,n+j\right]"$ denotes the context value of FM of the $\:"{C}^{th}"$ channel at position $\:"[i,j]"$. Now the pointwise context convolution$\:\:"{PC}_{j}"$ is done for $\:"{FM}_{CV}^{C}[m,n]"$ for adjusting the number of channels as in Eq. (53)

53 $$\:{PC}_{j}={\sum\:}_{i=1}^{C}{DK}_{ij}^{C}{FM}_{CV}^{C}[m,n,i]$$

The operation performed on PAW of original scale FM is formulated as in Eq. (54). The pointwise position convolution$\:\:"{PP}_{j}"$ is done for $\:"{FM}_{PV}^{C}[m,n]"$ for adjusting the number of channels as in Eq. (55). The value $\:"{FM}_{PV}^{C}[m,n]"$ represents the position value of the $\:"{C}^{th}"$ channel at position $\:"[m,n]"$. The $\:"{FMO}_{Pos}^{C}\left[m+i,n+j\right]"$ denotes the position value of FM of the $\:"{C}^{th}"$ channel at position $\:"[i,j]"$.

54 $$FM_{{PV}}^{C} [m,n] = \sum\nolimits_{{i = 1}}^{M} {\sum\nolimits_{{j = 1}}^{N} {DK_{{ij}}^{C} FMO_{{Pos}}^{C} [m + i,n + j]} }$$

55 $$\:{PP}_{j}={\sum\:}_{i=1}^{C}{DK}_{ij}^{C}{FM}_{PV}^{C}[m,n,i]$$

Now after obtaining the pointwise context convolution$\:\:"{PC}_{j}"$ of small-scale and pointwise position convolution$\:\:"{PP}_{j}"$ of original scale, both are subjected to batch normalization. Now let us consider the position block 1$\:\:"{FMO}_{PB1}"$ as denoted in Eq. (56). The convolution with 1 × 1 kernel was performed as denoted by Eq. (57). The value $\:\gamma\:$ denotes scaling factor, $\:\mu\:\:$denotes the mean of the position block 1, $\:{\sigma\:}^{2}\:$denotes the variance of the position block 1 and $\:\beta\:$ denotes the offset value. The final FM obtained from the position block 1 is$\:\:"{FMO}_{conv\_pos1}"$

56 $$FMO_{{PB1}} = \gamma \frac{{PP_{j} - \mu }}{{\sqrt {\sigma ^{2} + \in } }} + \beta$$

57 $$FMO_{{conv\_pos1}} = \sum\nolimits_{{i = 1}}^{M} {\sum\nolimits_{{j = 1}}^{N} D } K_{{ij}}^{C} FMO_{{PB1}} [i,j]$$

Now let us consider the context block 1$\:\:"{FMS}_{CB1}"$ as denoted in Eq. (58). The convolution with 1 × 1 kernel along with sigmoid activation function $\:"\sigma\:"$ was performed as denoted by Eq. (59) and Eq. (60). The final FM obtained from the context block 1 is$\:\:"{FMS}_{conv\_con1}"$.

58 $$FMS_{{CB1}} = \gamma \frac{{PC_{j} - \mu }}{{\sqrt {\sigma ^{2} + \in } }} + \beta$$

59 $$FMS_{{conv}} = \sum\nolimits_{{i = 1}}^{1} {\sum\nolimits_{{j = 1}}^{1} {DK_{{ij}}^{C} FMS_{{CB1}} [i,j]} }$$

60 $$\:{FMS}_{conv\_con1}=\sigma\:\left({FMS}_{conv}\right)$$

The SAW of original scale is denoted by $\:"{FMO}_{Con}"$ that denotes the “context block 2”. The $\:"{FMO}_{Con}"$and$\:\:"{FMS}_{Pos}"\:$is denoted as in Eq. (61) and Eq. (62). The PAW of small-scale FM is denoted by $\:"{FMS}_{Pos}"$ that denotes the “position block 2”.

61 $$\:{FMO}_{Con}\in\:{FM}^{H\times\:W\times\:C}$$

62 $$\:{FMS}_{Pos}\in\:{FM}^{\frac{H}{4}\times\:\frac{W}{4}\times\:C}$$

The SAW of the original scale FM is processed with convolution using 3 kernels as shown in Eq. (63). Now the pointwise context convolution$\:\:"{PWC}_{j}"$ is done for $\:"{\text{S}\text{A}\text{W}}_{CV}^{C}[m,n]"$ for adjusting the number of channels as in Eq. (64)

63 $$SAW_{{CV}}^{C} [m,n] = \sum\nolimits_{{i = 1}}^{2} {\sum\nolimits_{{j = 1}}^{2} {\sum\nolimits_{{k = 1}}^{C} D } } K_{{k,j,i}}^{C} FMO_{{Con}}^{C} [m + i,n + j]$$

64 $$\:{PWC}_{j}={\sum\:}_{i=1}^{C}{DK}_{ij}^{C}{\text{S}\text{A}\text{W}}_{CV}^{C}[m,n,i]$$

Now after obtaining the pointwise context convolution$\:\:"{PWC}_{j}"$ of original scale of the “context block 2”, the batch normalization operation was performed. Now let us consider the context block 2$\:\:"{FMO}_{CB2}"$ as denoted in Eq. (65) and Eq. (66). Then the obtained $\:{"FMO}_{conv}"\:$was up sampled with sigmoid activation function to form SAW of original scale FM as in Eq. (67) and Eq. (68). The final FM obtained from the context block 2 is$\:\:"{FMO}_{conv\_con2}"$.

65 $$FMO_{{CB2}} = \gamma \frac{{PWC_{j} - \mu }}{{\sqrt {\sigma ^{2} + \in } }} + \beta$$

66 $$FMO_{{conv}} = \sum\nolimits_{{i = 1}}^{1} {\sum\nolimits_{{j = 1}}^{1} D } K_{{ij}}^{C} FMO_{{CB2}} [i,j]$$

67 $$\:{FMUP}_{conv}=4*Upsample\left({FMO}_{conv}\right)$$

68 $$\:{FMO}_{conv\_con2}=\sigma\:\left({FMUP}_{conv}\right)$$

The PAW of the small-scale FM$\:\:"{FMS}_{Pos}"$ is processed with convolution using 3 kernels and 2 strides as in Eq. (69). Now the pointwise position convolution$\:\:"{PPC}_{j}"$ is done for $\:"{\text{P}\text{A}\text{W}}_{CV}^{C}[m,n]"$ for adjusting the number of channels as in Eq. (70)

69 $$PAW_{{CV}}^{C} [m,n] = \sum\nolimits_{{i = 1}}^{2} {\sum\nolimits_{{j = 1}}^{2} {\sum\nolimits_{{k = 1}}^{C} D } } K_{{k,j,i}}^{C} FMS_{{Pos}}^{C} [m*(2 - 1) + i,n*(2 - 1) + j]$$

70 $$\:{PPC}_{j}={\sum\:}_{i=1}^{C}{DK}_{ij}^{C}{\text{P}\text{A}\text{W}}_{CV}^{C}[m,n,i]$$

Now after obtaining the pointwise position convolution$\:\:"{PPC}_{j}"$ of small-scale of the “position block 2”, the batch normalization operation was performed. Now let us consider the position block 2$\:\:"{FMS}_{PB2}"$ as denoted in Eqs. (71) and (72).

71 $$FMS_{{PB2}} = \gamma \frac{{PPC_{j} - \mu }}{{\sqrt {\sigma ^{2} + \in } }} + \beta$$

72 $$FMS_{{conv}} = \sum\nolimits_{{i = 1}}^{1} {\sum\nolimits_{{j = 1}}^{1} D } K_{{ij}}^{C} FMS_{{PB2}} [i,j]$$

Then the obtained $\:{"FMS}_{conv}"\:$was performed with average pooling $\:"{FM}_{AP}[m,n]"\:$using 3 kernels and 2 strides to form PAW of small-scale FM as in Eq. (73). The final small-scale FM obtained from the position block 2 is$\:\:"{FMS}_{conv\_pos2}"$ as in Eq. (74).

73 $$FM_{{AP}} [m,n] = \frac{1}{9}\sum\nolimits_{{i = 1}}^{3} {\sum\nolimits_{{j = 1}}^{3} F } MS_{{conv}} [m*2 + i,\:n*2 + j]$$

74 $$\:{FMS}_{conv\_pos2}=\:{FM}_{AP}[m,n]$$

The final FM of original scale and small scale of position block 1, context block 1, context block and position block 2 is shown in Eq. (75) to Eq. (78)

75 $$\:{FMO}_{conv\_pos1}=Optimized\:Original\:Scale\:position\:FM\:of\:position\:block\:1\:$$

76 $$\:{FMS}_{conv\_con1}\:=Optimized\:Small\:Scale\:Context\:FM\:of\:context\:block\:1\:$$

77 $$\:{FMO}_{conv\_con2}=Optimized\:Original\:Scale\:Context\:FM\:of\:context\:block\:2\:$$

78 $$\:{FMS}_{conv\_pos2}=Optimized\:Small\:Scale\:\:position\:FM\:of\:position\:block\:2\:$$

The output feature map of original scale is $\:{FM}_{OS\_Out}\:$denoted in Eq. (79) and the output feature map of small-scale is $\:{FM}_{SS\_Out}$ denoted in Eq. (80).

79 $$\:{FM}_{OS\_Out}={FMO}_{conv\_pos1}\times\:{FMO}_{conv\_con2}$$

$\:{FM}_{SS\_Out}={FMS}_{conv\_con1}\times\:{FMS}_{conv\_pos2}$ (80) The optimized fused feature map is $\:{"OFM}_{Out}"$ obtained by combining the $\:{FM}_{OS\_Out}$ and $\:{FM}_{SS\_Out}$ as in Eq. (81). The “$\:Upsample$” operation resizes the lower-resolution FM $\:F{M}_{\left(S{S}_{Out}\right)}$to match the dimensions of $\:F{M}_{\left(O{S}_{Out}\right)}$, and the scaling factor (×4) ensures the intensities or channel magnitudes are balanced before fusion. The addition $\:"+"$ operation merges these two maps to form the final output feature map$\:\:"{OFM}_{Out\:}"$

81 $$\:{OFM}_{Out}={FM}_{OS\_Out}+4*Upsample\left({FM}_{SS\_Out}\right)$$

## Experimental setup and result analysis

**Fig. 8:**
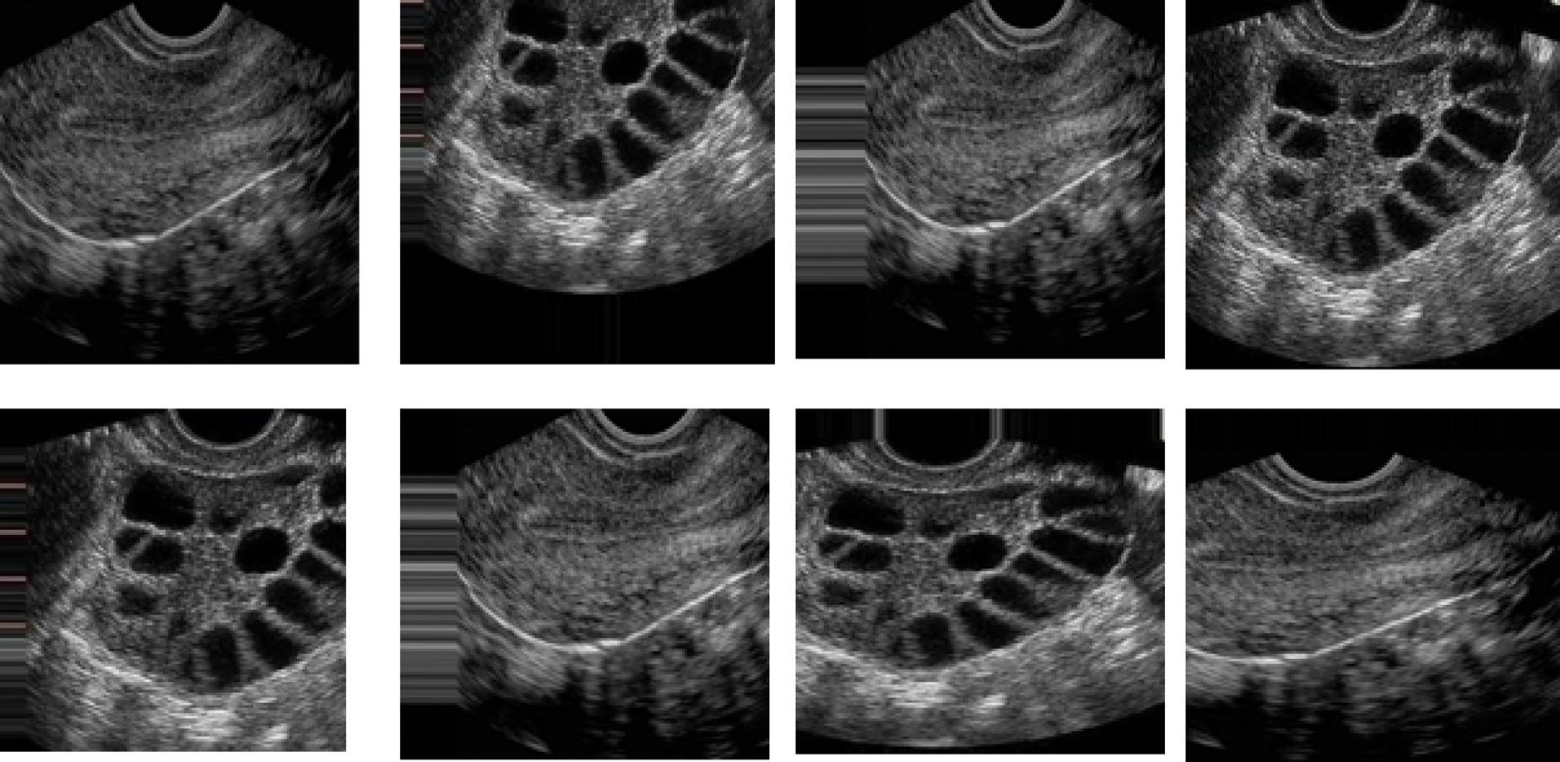
Sample PCOS Ultrasound Dataset Images.

**Fig. 9:**
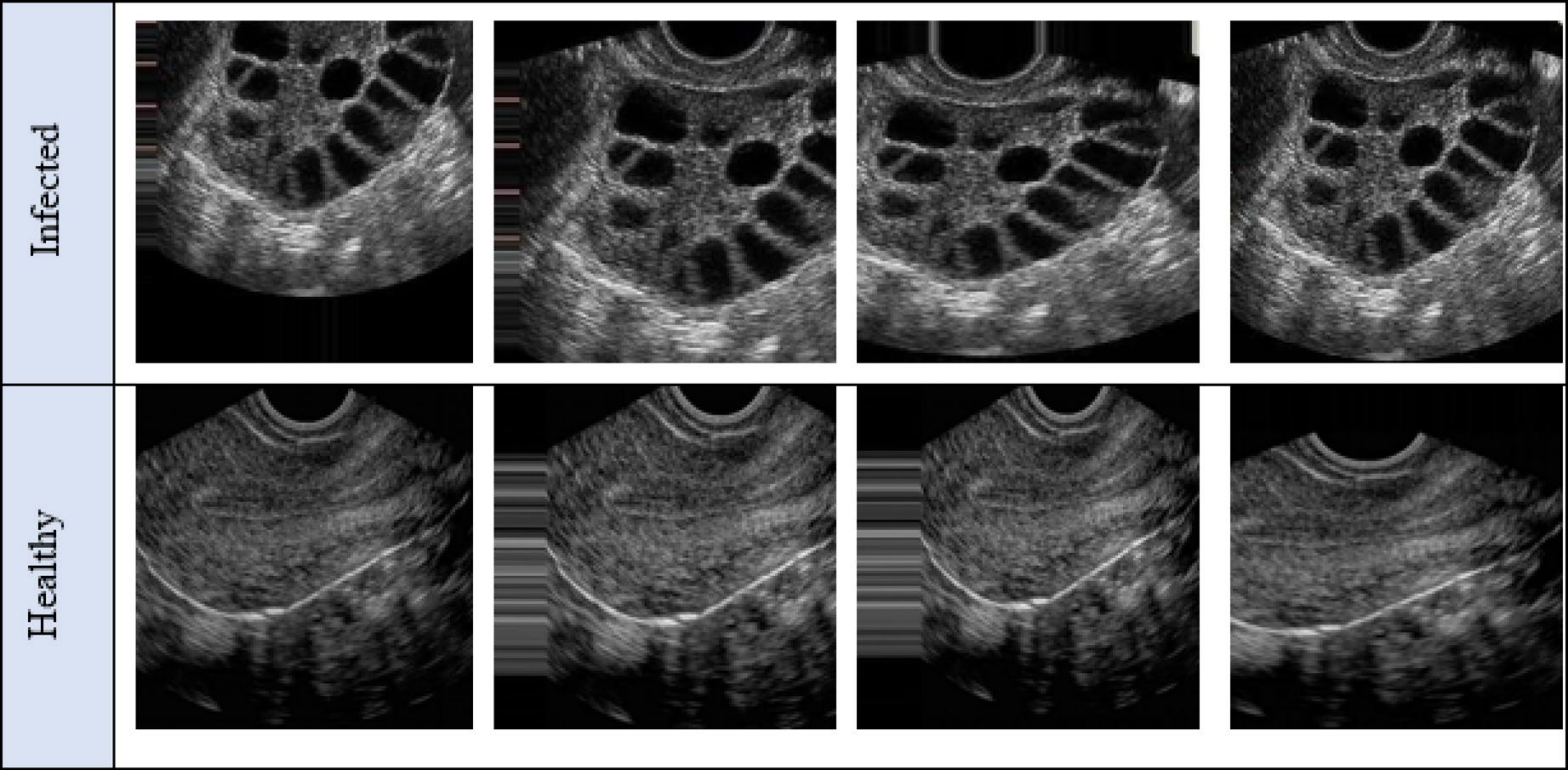
Results of Labeled PCOS Ultrasound Dataset Images.

The FCAU-Net initiates by collecting have 3800 ultrasound ovary images of PCOS Ultrasound (https://www.kaggle.com/datasets/anaghachoudhari/pcos-detection-using-ultrasound-images) dataset for classifying the PCOS infected and healthy class type. The sample normal and PCOS infected images from the dataset are shown in the Fig. 8. The implementation was carried out in python by using keras, tensorflow, pandas, numpy, algorithms, utils, skimage, neupy, matplotlib and Theano library. The PCOS Ultrasound from dataset are segregated based on the disease class. The segregated PCOS Ultrasound are performed with labeling and the results are shown in Fig. 9.

**Fig. 10:**
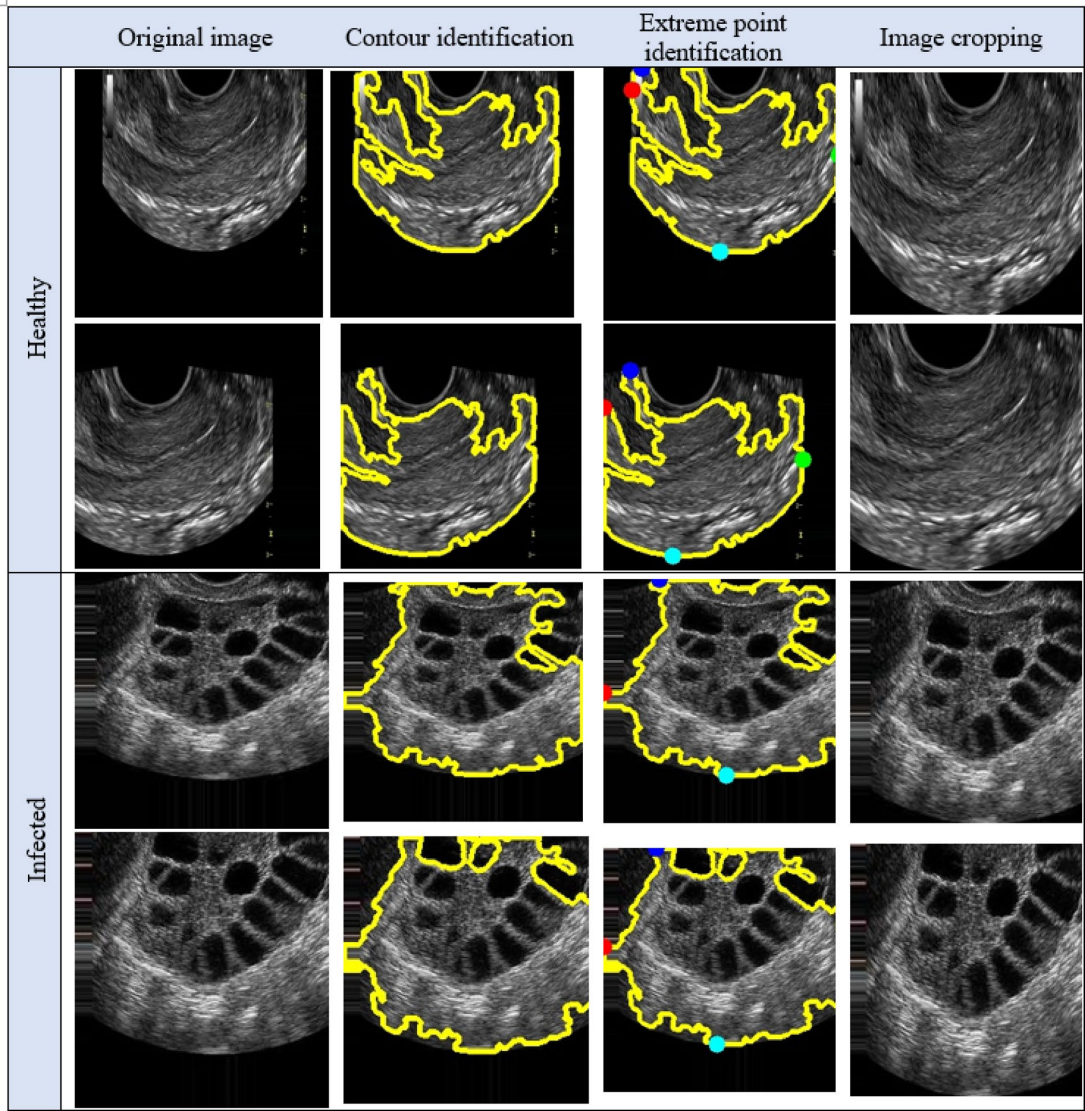
Step by step breakdown of cropping the image.

**Fig. 11:**
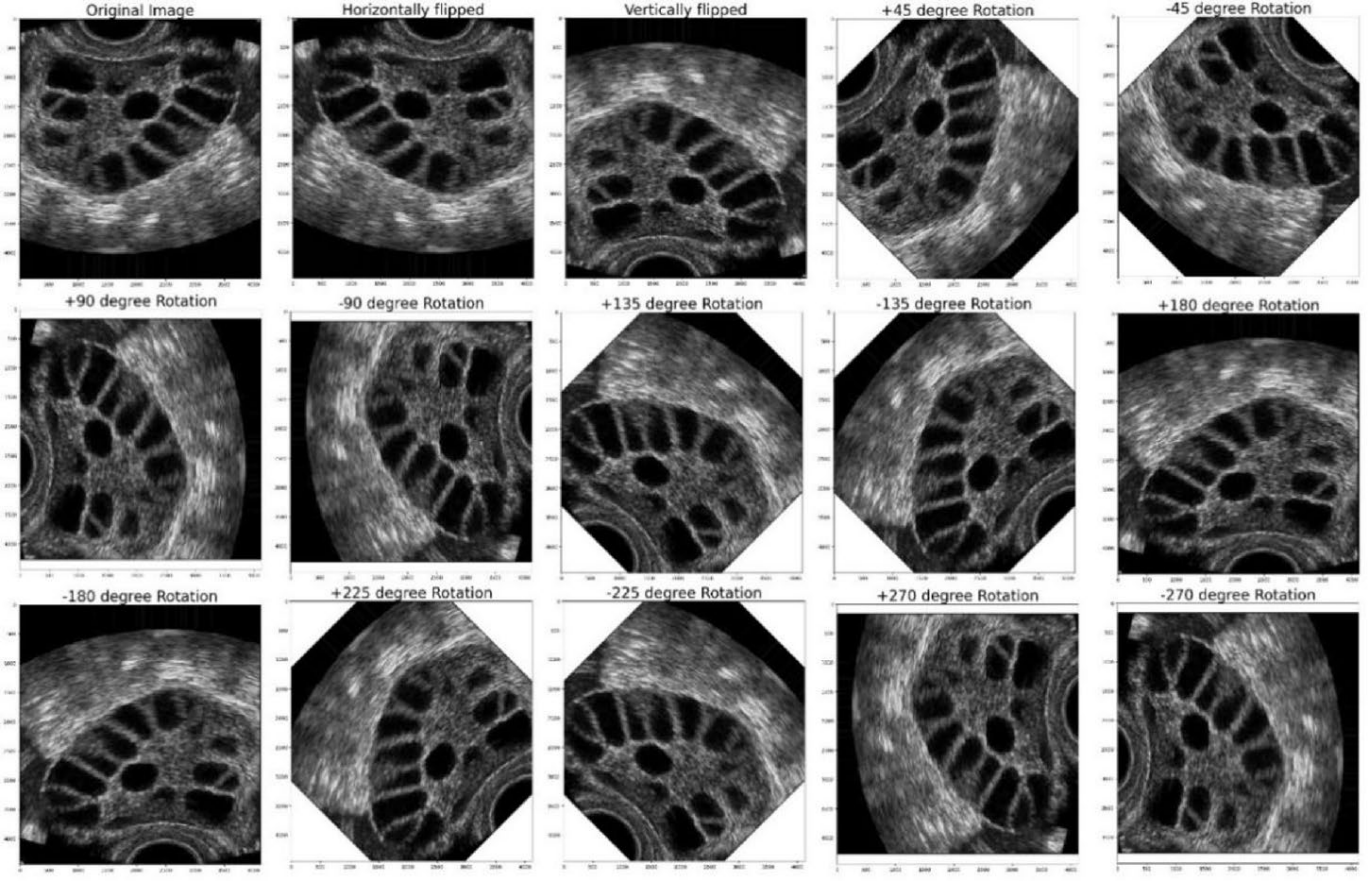
Results of PCOS infected Data Augmentation Images.

**Fig. 12:**
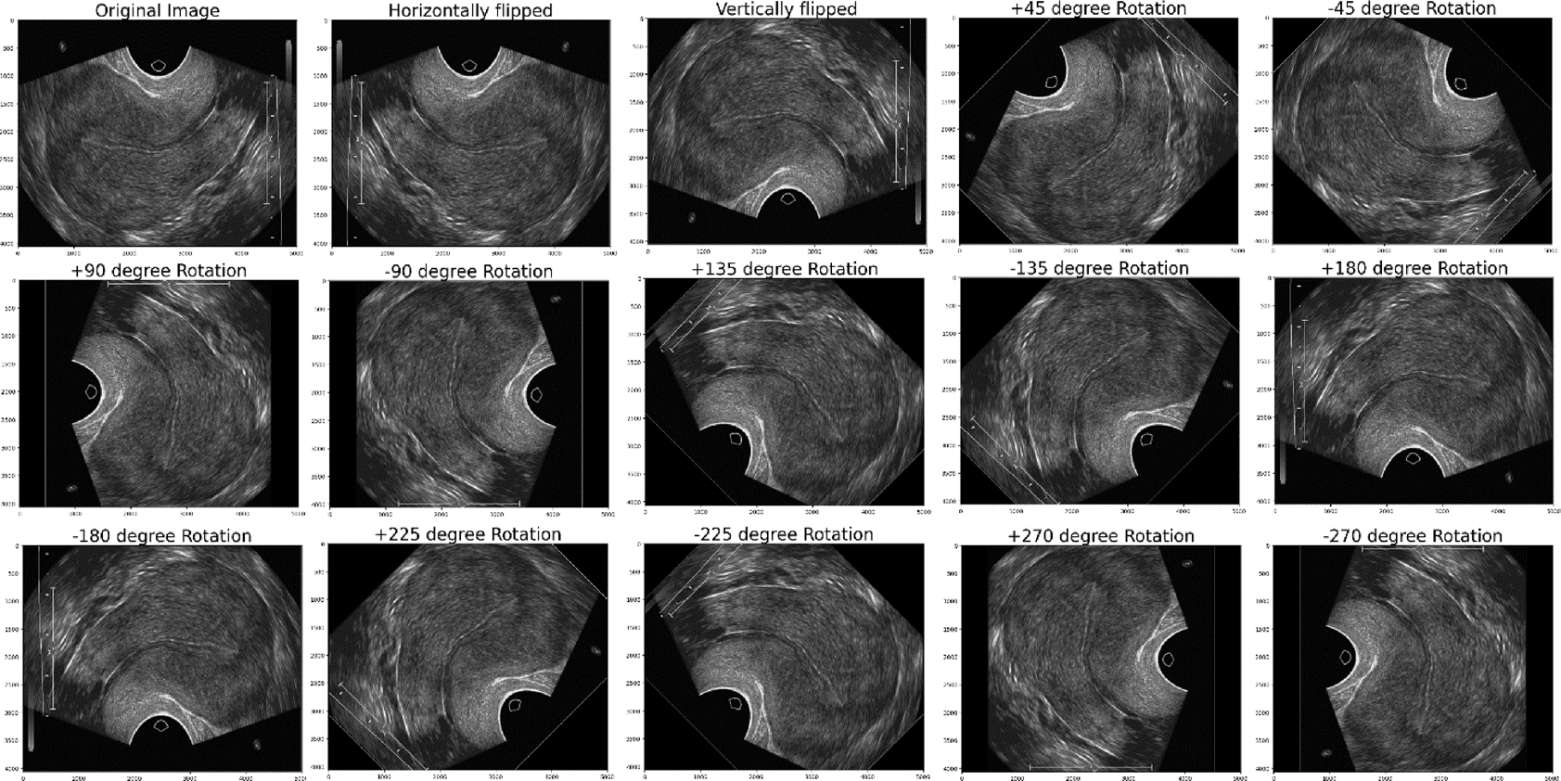
Results of Normal healthy ultrasound Data Augmentation Images.

After labeling, the image cropping was done to form the cropped image. The results of the PCOS Ultrasound images before and after Image cropping are shown in Fig. 10. The cropped PCOS Ultrasound images are subjected to data augmentation to form 14 images for each image resulting with 53,200 images data augmented cropped images. The results obtained from the data augmented PCOS infected and normal healthy ultrasound images are shown in Figs. 11 and 12 respectively.

The original dataset consisted of 3,800 ultrasound images with 1,900 healthy and 1,900 PCOS-infected. The testing dataset was formed with 80: 20 to extract 360 testing images. Through augmentation techniques, the dataset was expanded to 42,560 images, resulting in a total of 45,600 images. Before performing the augmentation process, the dataset was initially divided into separate subsets for training and testing in the ratio of 80:20 to ensure unbiased model evaluation. Specifically, the testing dataset was formed exclusively from the original, unaltered ultrasound 360 images prior to any augmentation procedures. This approach guarantees that the test data remains completely independent from the augmented samples used during model training, thereby preserving the integrity of performance assessment. Once the testing set was isolated, data augmentation was applied only to the training subset to artificially expand the number and diversity of training samples. The augmentation operations were implemented to simulate realistic variations in ultrasound imaging conditions. By using augmented data solely for training, the FCAU-Net model benefits from improved generalization and robustness to image variability, while the testing phases are conducted strictly on original data to ensure an accurate reflection of the model’s real-world diagnostic performance. This methodological separation between augmentation and testing provides a clear, reliable framework for evaluating model effectiveness without data leakage or overfitting bias. Table 3 summarizes the distribution of PCOS classes across the augmented datasets. The large augmented dataset not only enhances the model’s generalization capability but also reduces the risk of overfitting, ensuring that the proposed FCAU-Net can robustly distinguish PCOS from healthy cases.

**Table 3:** Dataset distribution for FCAU-Net training, validation and testing.

	Data distribution						
PCOS class	Actual	Testing	Actual	Augmentation	Total	Train	Validation
Healthy	1,900	380	1,520	21,280	22,800	18,240	4560
PCOS infected	1,900	380	1,520	21,280	22,800	18,240	4560
Total	3,800	760	3040	42,560	45,600	36,480	9120

The brightness estimation for Histogram equalized images, CLAHE images and FCE images was done and its analysis is shown in Table. 4. The brightness was analyzed for some sample images from the PCOS ultrasound image dataset. From the Table. 4, it is evident that the brightness value of FCE images was found to be high compared to other two methods. From the Table. 4, it is evident that while HE and CLAHE produce only slight improvements in brightness typically within 1 to 3% of each other, the FCE method significantly outperforms both, achieving brightness values consistently above 94%. This sharp increase indicates that FCE not only enhances the global contrast but also preserves finer structural details, making ovarian features more distinguishable. Importantly, both PCOS-infected and healthy images benefit equally from FCE, showing a consistent improvement trend, which implies that the technique is effective. Such a substantial enhancement in brightness directly supports improved feature visibility, thereby facilitating better feature extraction and classification performance.

**Table 4.** Brightness analysis of HE, CLAHE and FCE images.

S. NO	Sample Image	Brightness Value (%)		
		HE Images	CLAHE Images	FCE Images
	PCOS Infected Image 1	61.43	64.44	96.43
	PCOS Infected Image 2	62.33	63.32	94.63
	PCOS Infected Image 3	63.63	65.61	95.63
	PCOS Infected Image 4	67.22	68.21	99.22
	PCOS Infected Image 5	65.66	66.34	97.66
	Healthy ultrasound Image 1	66.34	67.32	96.34
	Healthy ultrasound Image 2	61.55	64.22	97.55
	Healthy ultrasound Image 3	62.35	63.44	98.35
	Healthy ultrasound Image 4	63.45	65.46	97.45
	Healthy ultrasound Image 5	66.23	68.52	96.23

**Fig. 13:**
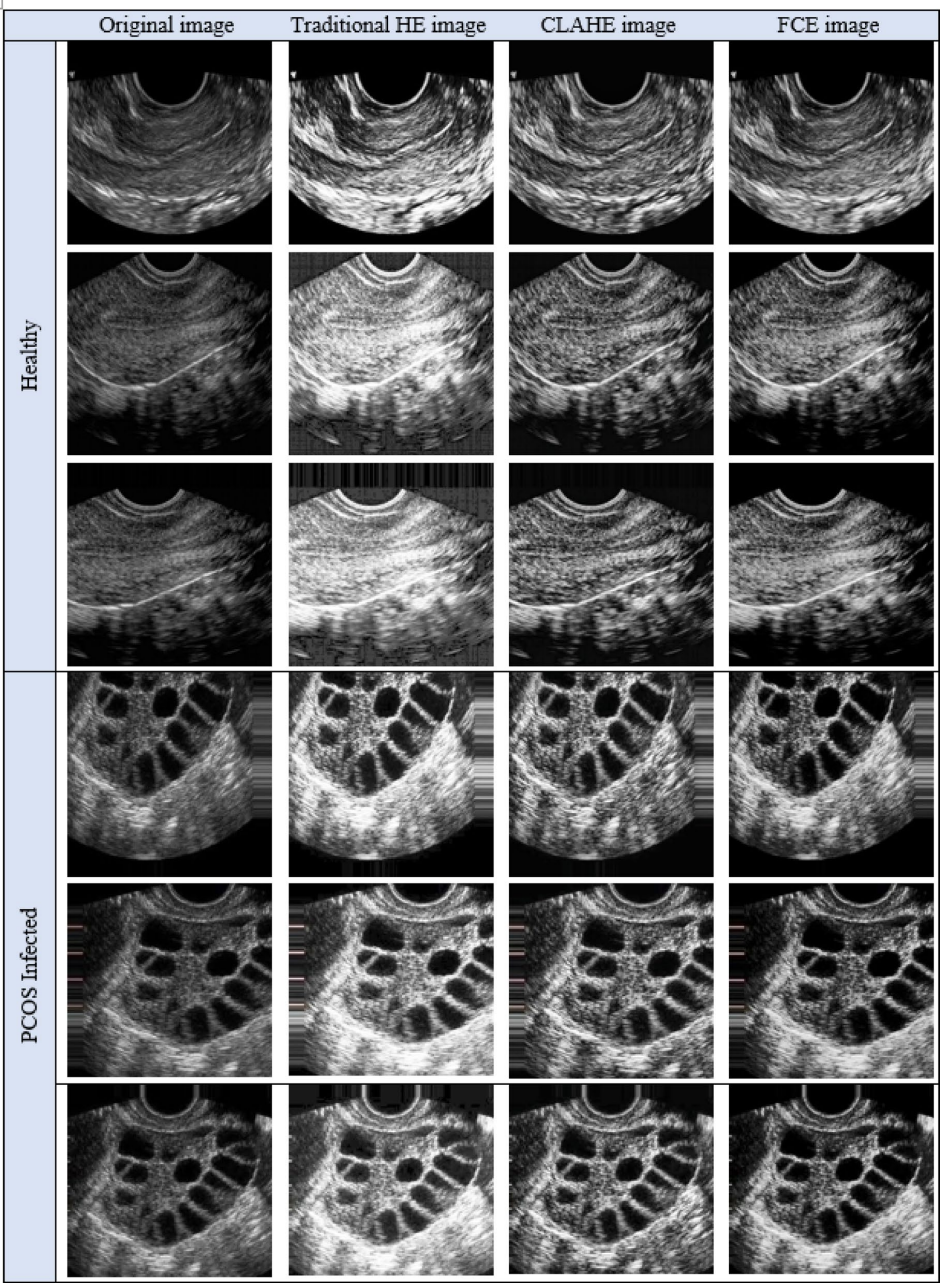
Results of Traditional HE, CLAHE and FCE images of both classes.

The Data augmentation images are subjected to form the Histogram equalized images, CLAHE images and FCE images and the obtained results are shown in Fig. 13.

**Fig. 14:**
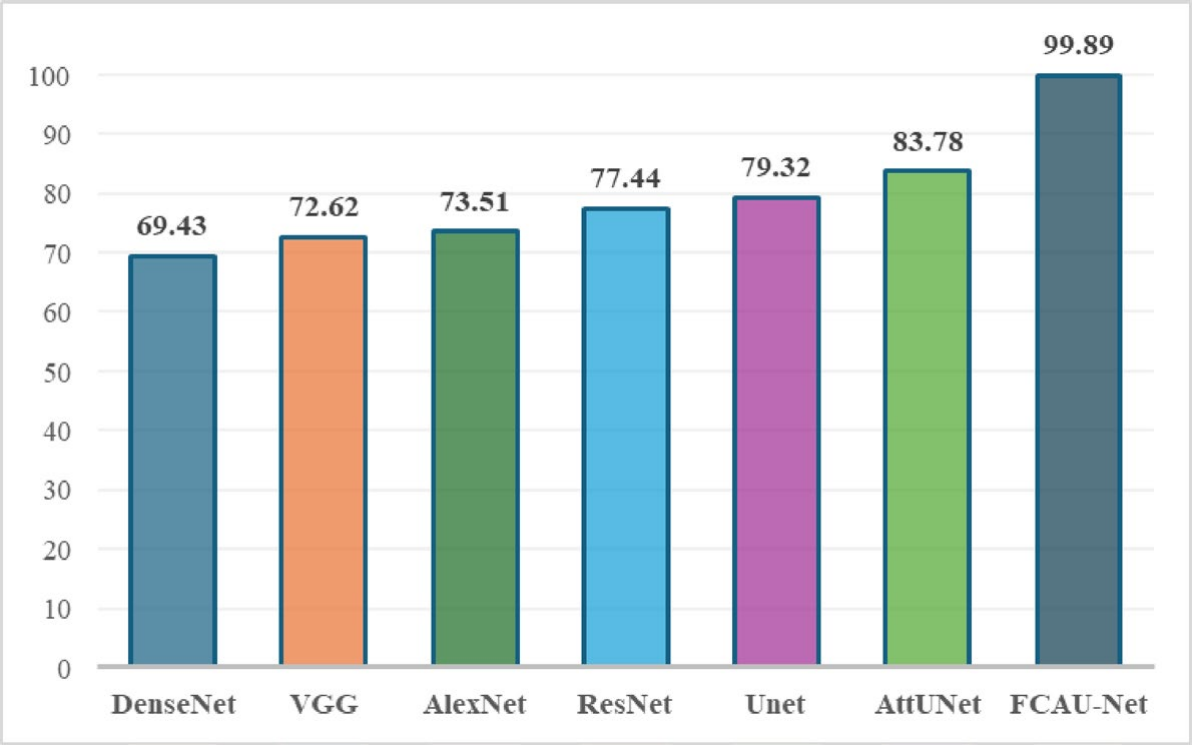
Performance analysis of Accuracy of FCAU-Net.

The training images was fitted with proposed FCAU-Net and tested with the existing CNN to analyze the performance and is shown in Table 5; Fig. 14. It is observed that Attention U-Net was found to exhibit the accuracy above 80%. So, the Attention U-Net was refined by integrating the feature fusion context module to propose FCAU-Net model. To further validate the classification capability of the proposed FCAU-Net model, the confusion matrix was generated for the test dataset.

**Table 5:** Performance analysis of FCAU-Net.

CNN Type	Accuracy	
	Directly applying raw images	**Applying FCE images**
DenseNet^48–50^	68.27	69.43
VGG^51,52^	71.23	72.62
AlexNet^53^	72.91	73.51
ResNet^54,55^	76.25	77.44
U-Net^56^	78.64	79.32
Attention U-Net^57^	82.36	83.78
Proposed FCAU-Net	90.51	99.89

**Fig. 15:**
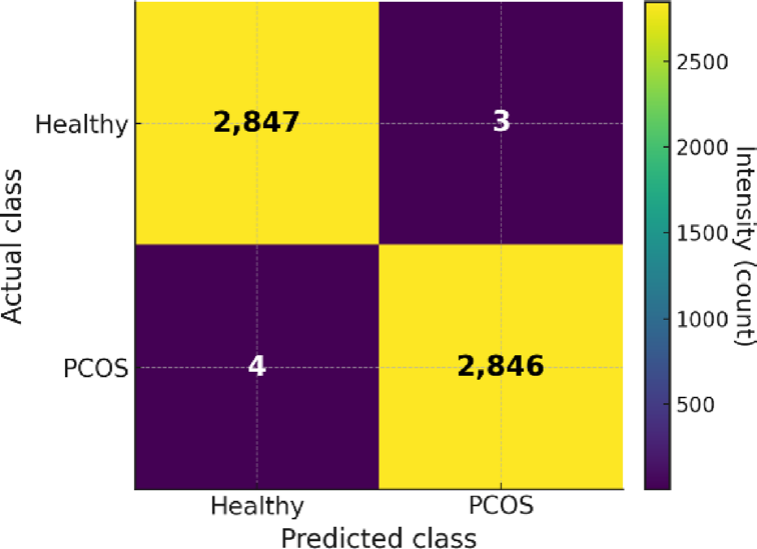
Confusion matrix of the proposed FCAU-Net.

**Fig. 16:**
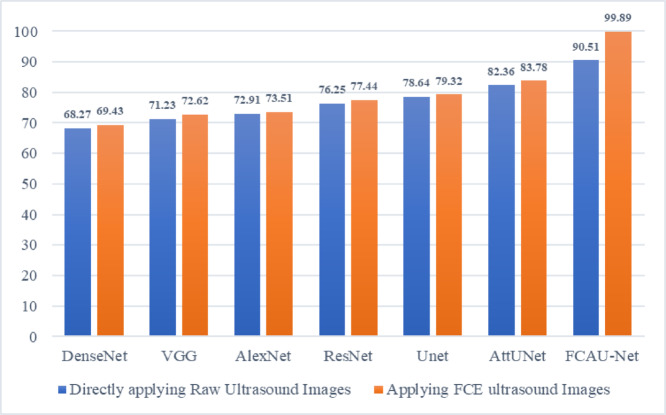
Performance analysis of Accuracy of FCAU-Net with and without applying FCE.

**Fig. 17:**
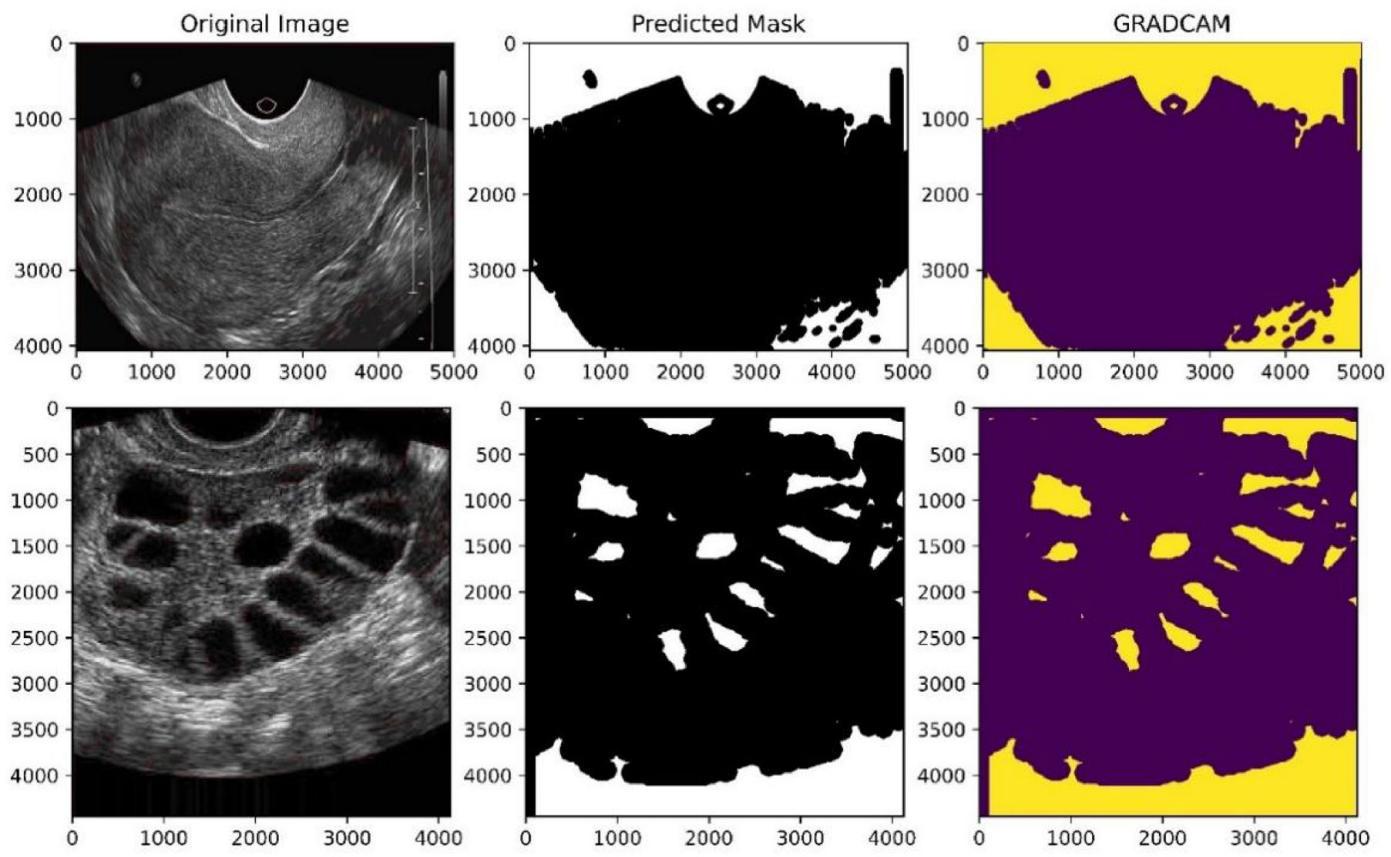
Prediction Mask Results of Proposed FCAU-Net.

The confusion matrix provides a detailed breakdown of correctly and incorrectly classified samples across the two classes healthy and PCOS-infected. As shown in Figure. 15, FCAU-Net demonstrates near-perfect classification performance, correctly identifying the vast majority of cases with only a very small number of misclassifications. Out of 5,700 test images, the model achieved 5,693 correct predictions, with just 7 errors, corresponding to an overall accuracy of 99.89%. From the Fig. 15, it is evident that both Healthy and PCOS classes are classified with almost equal precision, ensuring the model does not suffer from class imbalance bias. The prediction results obtained from proposed FCAU-Net is shown in Figs. 16 and 17.

To quantitatively assess the alignment of Grad-CAM heatmaps with ground-truth masks, four explainability metrics were used to analyze the performance like Intersection over Union (IoU), Dice Similarity Coefficient (DSC), Pointing Game Accuracy (PGA), and Energy-based Localization Score (ELS) and is shown in Table 6. IoU measures the overlap between the predicted Grad-CAM heatmap region P and the ground-truth mask G. Here $\:\left|\text{P}\text{∩}\text{G}\right|$is the number of pixels common to both prediction and ground truth, and $\:\left|\text{P}\text{∪}\text{G}\right|$ is the total number of unique pixels. The DSC evaluates the similarity between predicted and ground-truth regions, emphasizing balanced overlap where $\:\left|\text{P}\right|$ and $\:\left|\text{G}\right|$denote the number of pixels in the predicted region and ground truth, respectively. PGA assesses whether the most activated pixel from the Grad-CAM heatmap lies within the ground-truth region. ELS evaluates the proportion of activation energy concentrated within the ground-truth region compared to the total energy in the heatmap.

**Table 6:** Key metrics of quantitative evaluation of Explainability.

Metrics	Formula
IoU	${\text{IoU = }}\frac{{\left| {{\text{P}} \cap {\text{G}}} \right|}}{{\left| {{\text{P}} \cup {\text{G}}} \right|}}$
DSC	${\text{DSC}} = \frac{{2\left| {{\text{P}} \cap {\text{G}}} \right|}}{{\left| {\text{P}} \right| + \left| {\text{G}} \right|}}$
PGA	$\overline{{{\text{Var}}}} = \frac{{{\text{No}}{\text{.}}\:{\text{of}}\:{\text{hits}}}}{{{\text{Total}}\:{\text{samples}}}} \times 100$
ELS	${\text{SD}} = \frac{{\sum\nolimits_{{i \in G}} {{\text{GRADCAM}}_{i} } }}{{\sum\nolimits_{{i \in I}} {{\text{GRADCAM}}_{i} } }} \times 100$

The numerical assessment of GRAD-CAM performance is shown in Table 7. The Table 7 performance analysis provides a numerical assessment of how well the highlighted regions in Grad-CAM align with the ovarian regions. The proposed FCAU-Net consistently demonstrates higher alignment scores compared to baseline CNN models, reinforcing that the attention learned by FCAU-Net is both accurate and meaningful.

**Table 7:** Quantitative evaluation of explainability Grad-CAM of FCAU-Net.

Model	IoU (%)	DSC (%)	PGA (%)	ELS (%)
DenseNet	58.12	62.45	67.21	64.38
VGG	60.27	64.88	69.14	65.02
AlexNet	61.33	65.74	70.29	66.41
ResNet	66.41	70.83	74.56	71.22
U-Net	72.89	76.12	80.35	78.64
Attention U-Net	78.25	82.46	86.14	83.71
FCAU-Net	92.67	95.03	97.81	95.42

**Fig. 18:**
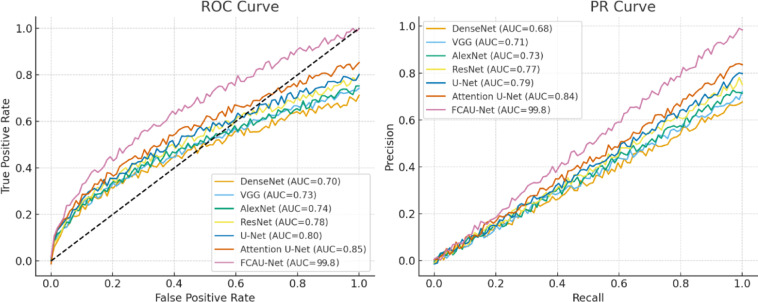
ROC curve and PR curve of Proposed FCAU-Net.

From the quantitative analysis of Table 7, it is evident that FCAU-Net achieves the highest explainability scores across all metrics. Specifically, the DSC (95.03%) and IoU (92.67%) indicate that Grad-CAM heatmaps produced by FCAU-Net strongly overlap with the true follicular regions in the ultrasound images. Furthermore, the PGA (97.81%) highlights that FCAU-Net almost always localizes the clinically relevant region, while the high ELS (95.42%) reflects the models focus intensity on these target areas. Compared to conventional CNNs and even the Attention U-Net, FCAU-Net shows a substantial margin of improvement, validating that its FFCM module not only enhances classification accuracy but also improves interpretability in meaningful way. Fig. 18 shows the ROC curve and PR curve of the proposed FCAU-Net and it almost reaches the top-left corner in ROC and near-perfect PR, reflecting its very high accuracy of 99.89% on FCE images. PR curves show how the proposed FCAU-Net maintains high precision even at high recall, critical for reducing false positives in PCOS diagnosis.

## Cross-validation generalization performance on FCAU-Net

**Fig. 19:**
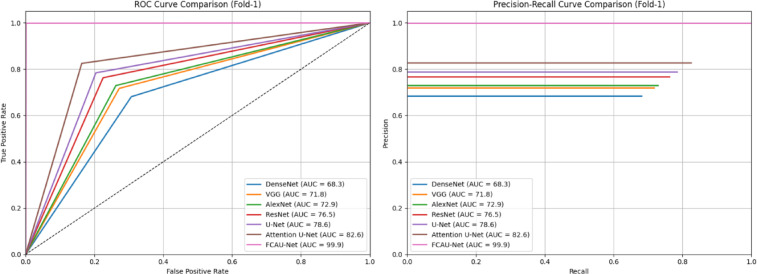
ROC curve and PR curve of FCAU-Net Fold-1.

To further validate the robustness and generalization ability of the proposed FCAU-Net, the 5-fold cross-validation was conducted on the augmented PCOS images. Unlike a single train–test split, k-fold cross-validation systematically partitions the dataset into k equally sized folds, where in each iteration one-fold is used for testing and the remaining k-1 folds for training. This process is repeated until every fold has been used once as the test set. The final performance is obtained by averaging across all folds, ensuring that the reported results are not biased by a particular train–test division. So, k-fold cross-validation ensures that every sample in the dataset is used for both training and testing, thereby minimizing bias and reducing the risk of overfitting. By systematically rotating training and testing folds, the performance of FCAU-Net can be reliably assessed under different data partitions. For the k-fold cross-validation experiments, the augmented PCOS 45,600 images with 22,800 Healthy and 22,800 PCOS images. The dataset was partitioned into five equal folds, each containing 9120 images, ensuring that every sample contributed to both training and testing across different iterations. In each fold, approximately 45,600 images were used for training while the 360 actual images were used for testing. Table 8 shows the performance of Fold-1 of cross-validation results. The results of Fold-1 demonstrate that the proposed FCAU-Net outperforms all baseline CNN architectures across every evaluation metric. However, the proposed FCAU-Net clearly establishes superiority, reaching nearly perfect performance in Fold 1 with accuracy and F1-scores above 99%. This fold-1 thus validates the effectiveness of the FFCM module in capturing both positional and contextual information, results in a more reliable diagnostic tool. The ROC and PR curves for Fold-1 in Figure. 19 shows that FCAU-Net significantly outperforms all other models, achieving near-perfect classification.

**Table 8:** Performance analysis of fold-1 cross-validation.

Model	Accuracy	Precision	Recall	Specificity	F1-Score
DenseNet	68.9	68.5	68.1	69.3	68.3
VGG	72.2	71.9	71.7	72.8	71.8
AlexNet	73.4	73.0	72.9	73.8	72.9
ResNet	77.0	76.7	76.3	77.5	76.5
U-Net	79.2	78.9	78.4	79.6	78.6
Attention U-Net	83.1	82.7	82.5	83.7	82.6
FCAU-Net	99.9	99.9	99.8	99.9	99.9

Table 9 shows the performance of Fold-2 of cross-validation results. In Fold 2, the performance trend observed in Fold 1 is consistently replicated. Nevertheless, FCAU-Net exhibits the most robust performance across all metrics, maintaining balanced precision, recall, specificity, and F1-scores above 99%. The high specificity indicates that the model effectively reduces false positives, avoiding over-diagnosis of healthy ovaries as PCOS. This balance between sensitivity and specificity is vital, where both false negatives and false positives carry serious consequences. This fold-2 reinforces the generalization ability of FCAU-Net, suggesting that its superior accuracy is not dataset-specific but consistent across splits.

**Table 9:** Performance analysis of fold-2 cross-validation.

Model	Accuracy	Precision	Recall	Specificity	F1-Score
DenseNet	69.4	68.9	68.6	70.1	68.7
VGG	72.7	72.2	72.0	73.3	72.1
AlexNet	73.8	73.4	73.1	74.2	73.2
ResNet	77.5	77.2	76.8	78.0	77.0
U-Net	79.5	79.1	78.8	79.9	78.9
Attention U-Net	83.6	83.3	82.9	84.1	83.1
FCAU-Net	99.8	99.8	99.7	99.9	99.8

**Fig. 20:**
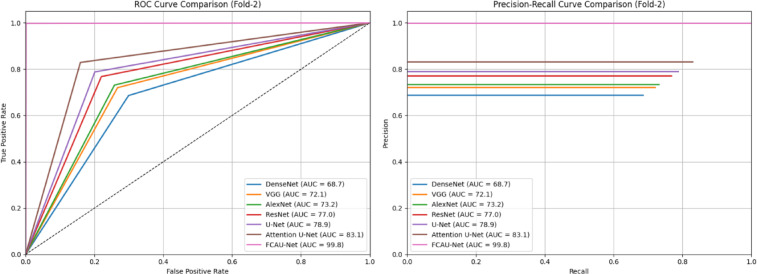
ROC curve and PR curve of FCAU-Net Fold-2.

**Fig. 21:**
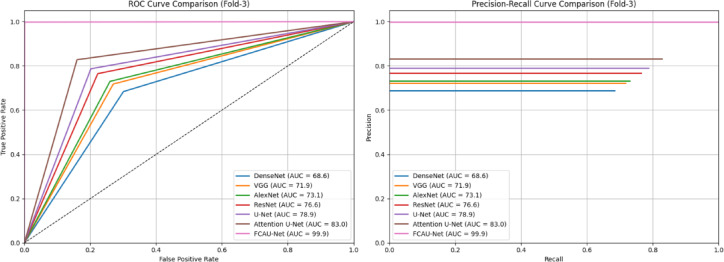
ROC curve and PR curve of FCAU-Net Fold-3.

The ROC and PR curves for Fold-2 in Fig. 20 shows that FCAU-Net significantly outperforms all other models, achieving near-perfect classification. The Fold-3 performance analysis of FCAU-Net shown in Table 10 further illustrates the robustness of FCAU-Net in comparison with other baseline models. FCAU-Net maintains its dominance, achieving near-perfect classification with F1-scores and recall emphasizes the model’s ability to correctly identify nearly all PCOS cases, ensuring minimal risk of underdiagnosis. Additionally, the stability of results across folds highlights that FCAU-Net does not overfit to a particular data partition. The ROC and PR curves for Fold-3 in Fig. 21 shows that FCAU-Net significantly outperforms all other models, achieving near-perfect classification.

**Table 10:** Performance analysis of fold-3 cross-validation.

Model	Accuracy	Precision	Recall	Specificity	F1-Score
DenseNet	69.2	68.8	68.4	69.9	68.6
VGG	72.5	72.1	71.8	73.0	71.9
AlexNet	73.6	73.3	73.0	74.0	73.1
ResNet	77.2	76.8	76.5	77.7	76.6
U-Net	79.4	79.1	78.7	79.8	78.9
Attention U-Net	83.5	83.2	82.8	84.0	83.0
FCAU-Net	99.9	99.9	99.8	99.9	99.9

The Fold-4 performance analysis of FCAU-Net shown in Table 11 confirms the FCAU-Net’s superiority. The proposed FCAU-Net achieves accuracies above 99% with correspondingly high precision and specificity, signifying a substantial reduction in both false positives and false negatives. Such stability across folds demonstrates that FCAU-Net is not only accurate but also highly generalizable for real time deployment.

**Table 11:** Performance analysis of fold-4 cross-validation.

Model	Accuracy	Precision	Recall	Specificity	F1-Score
DenseNet	69.6	69.2	68.7	70.2	68.9
VGG	72.9	72.4	72.2	73.4	72.3
AlexNet	74.0	73.7	73.3	74.4	73.5
ResNet	77.6	77.2	76.9	78.1	77.1
U-Net	79.6	79.2	78.9	80.0	79.0
Attention U-Net	83.7	83.4	83.0	84.2	83.2
FCAU-Net	99.9	99.9	99.8	99.9	99.9

**Fig. 22:**
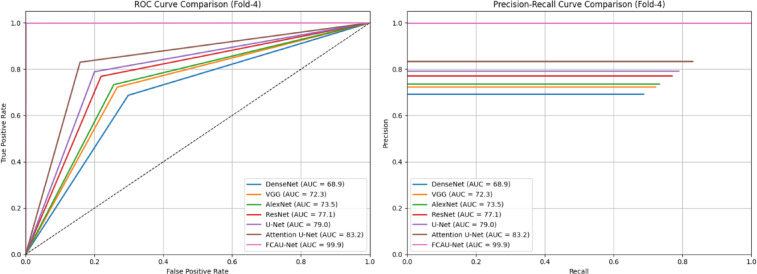
ROC curve and PR curve of FCAU-Net Fold-4.

The ROC and PR curves for Fold-4 in Fig. 22 shows that FCAU-Net significantly outperforms all other models, achieving near-perfect classification. The Fold-5 performance analysis of FCAU-Net shown in Table 12 results provide further evidence of FCAU-Net’s robustness, as the model consistently surpasses all baselines in every performance metric. While traditional CNNs, including DenseNet and AlexNet, remain constrained to accuracies below 75%, U-Net and ResNet achieve moderate improvements yet fall short in sensitivity.

**Table 12:** Performance analysis of fold-5 cross-validation.

Model	Accuracy	Precision	Recall	Specificity	F1-Score
DenseNet	69.1	68.6	68.3	69.7	68.5
VGG	72.4	72.0	71.7	72.9	71.8
AlexNet	73.5	73.1	72.9	73.9	73.0
ResNet	77.3	76.9	76.5	77.8	76.7
U-Net	79.3	79.0	78.6	79.7	78.8
Attention U-Net	83.4	83.0	82.7	83.9	82.9
FCAU-Net	99.8	99.8	99.7	99.9	99.8

**Fig. 23:**
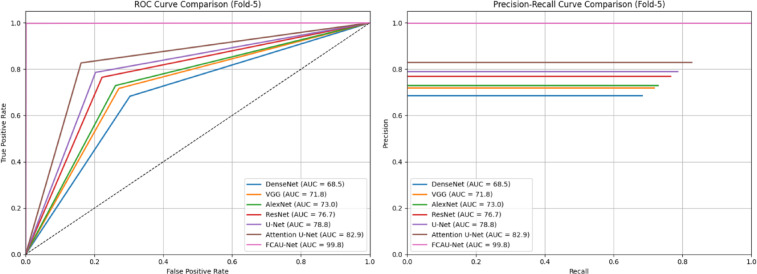
ROC curve and PR curve of FCAU-Net Fold-5.

**Fig. 24:**
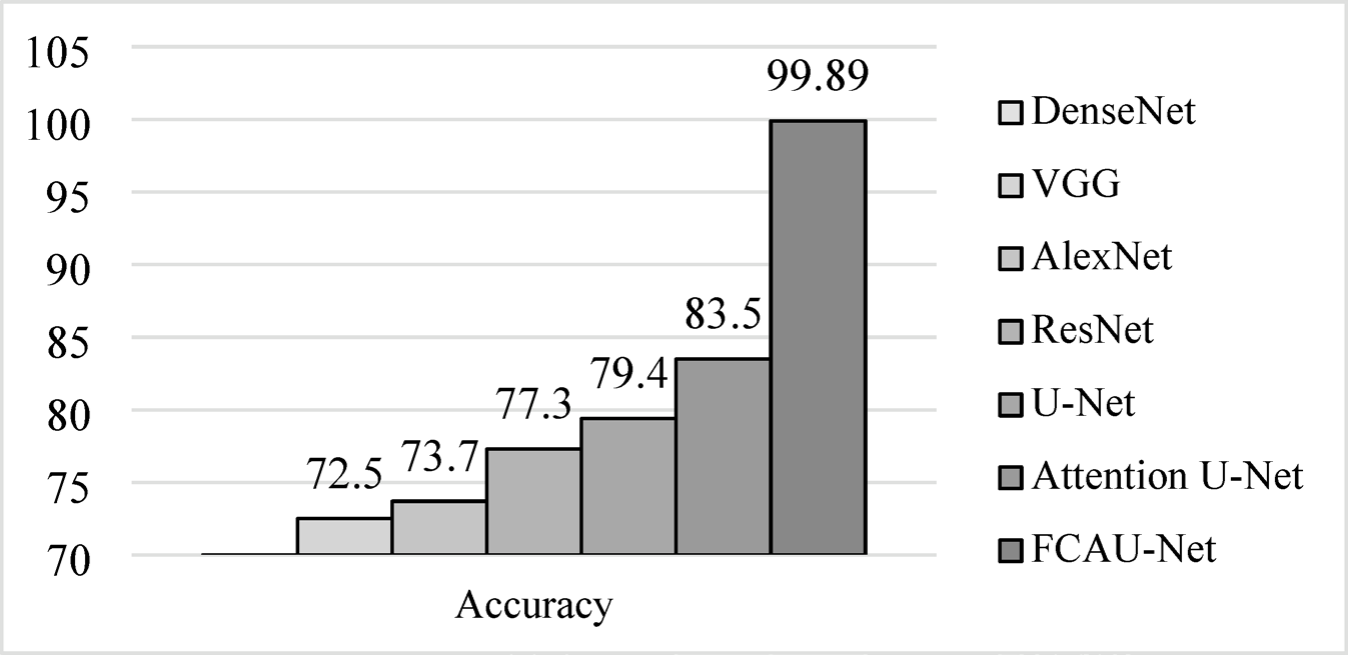
K-Fold cross validation plot of Proposed FCAU-Net.

**Fig. 25:**
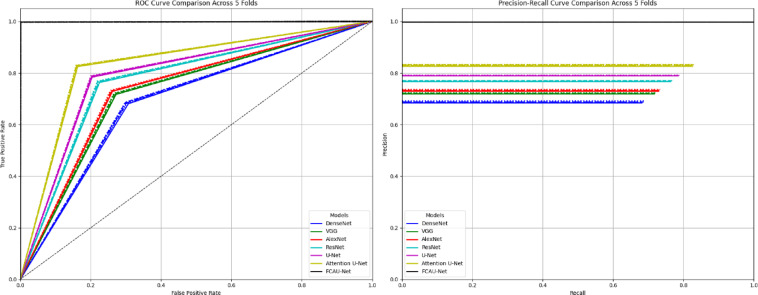
Consolidated ROC curve and PR curve of FCAU-Net K-fold cross validation.

The ROC and PR curves for Fold-5 in Fig. 23 shows that FCAU-Net significantly outperforms all other models, achieving near-perfect classification. Attention U-Net’s ability to surpass 83% accuracy highlights the incremental gains of attention mechanisms but also underscores the gap left unaddressed in terms of feature fusion. FCAU-Net addresses these limitations by achieving almost flawless classification, with balanced precision, recall, specificity, and F1-scores near 100%. This demonstrates its capacity to reliably capture both fine-grained local features and broader contextual dependencies within ovarian ultrasound images. The mean performance across all five folds provides the validation of FCAU-Net’s generalization capabilities and is shown in Table 13. Baseline models such as DenseNet, VGG, and AlexNet display consistently low averages across metrics, with accuracies around 70% to 74%. ResNet and U-Net improve average accuracy to the high 70%, but their lower recall values suggest persistent vulnerability to false negatives. Attention U-Net consistently achieves above 83% accuracy, marking a significant advancement through the use of attention mechanisms. However, FCAU-Net outperforms all models by a wide margin, with mean accuracies above 99% and near-perfect scores across precision, recall, specificity, and F1-score. This consistency across folds confirms that FCAU-Net’s results are not due to random partitioning effects but rather from its architecture, which integrates feature fusion and contextual attention. Figure 24 shows the 5-fold cross validation box plot of proposed FCAU-Net. The consolidated ROC and PR curves for all the five folds in Fig. 25 shows that FCAU-Net significantly outperforms all other models, achieving near-perfect classification.

**Table 13:** Performance analysis of fold-5 cross-validation.

Model	Accuracy	Precision	Recall	Specificity	F1-Score
DenseNet	69.2	68.8	68.4	69.8	68.6
VGG	72.5	72.1	71.9	73.1	72.0
AlexNet	73.7	73.3	73.0	74.1	73.1
ResNet	77.3	76.9	76.6	77.8	76.7
U-Net	79.4	79.0	78.7	79.8	78.9
Attention U-Net	83.5	83.1	82.8	84.0	83.0
FCAU-Net	99.89	99.9	99.8	99.9	99.9

## Statistical significance testing on FCAU-Net

To ensure that the superior performance of the proposed FCAU-Net with 99.89% is not due to random variation, the statistical significance testing was conducted. To evaluate the effectiveness of the proposed FCAU-Net model, a statistical significance analysis was conducted comparing its performance on raw ultrasound images and FCE enhanced ultrasound images. While the absolute accuracy of FCAU-Net increased from 90.51% to 99.89%, it is essential to determine whether this improvement is statistically meaningful. Accuracy alone cannot fully establish the robustness of a model, hence statistical significance tests was employed to validate the consistency of FCAU-Net compared with baseline models such as DenseNet, VGG, AlexNet, ResNet, U-Net, and Attention U-Net.For statistical comparison. Paired t-test was used to compare the performance of FCAU-Net with each baseline model. McNemar test was also conducted on confusion matrices to evaluate whether the observed differences in misclassification distributions were statistically significant. A 95% confidence level (*p* < 0.05) was set as the threshold for significance. Table 14 shows the performance of statistical significance testing analysis results on confusion matrix with FCE images.

**Table 14:** Statistical significance testing on confusion matrix with FCE images.

Model	With FCE images (%)				
	Accuracy	Precision	Recall	F1-score	Misclassifications
DenseNet	69.43	68.92	68.54	68.73	1,742
VGG	72.62	72.40	72.01	72.20	1,561
AlexNet	73.51	73.28	72.89	73.08	1,508
ResNet	77.44	77.20	76.92	77.06	1,286
U-Net	79.32	79.11	78.72	78.91	1,178
Attention U-Net	83.78	83.50	83.19	83.34	926
Proposed FCAU-Net	99.89	99.89	99.86	99.87	7

The statistical paired t-test was employed to assess the differences in model performance, considering repeated measurements across multiple runs on raw ultrasound images and FCE ultrasound images. Key metrics computed include the mean difference, variance, standard deviation, t-value, degrees of freedom (DoF), *p*-value, and 95% confidence interval of the observed improvement and is shown in Table 15.

**Table 15:** Key metrics of statistical significance testing.

Metrics	Formula
Accuracy difference$\:{\text{}\text{Diff}}_{\text{i}}$	${\text{Diff}}_{i} = {\text{Accuracy}}\;({\text{FCE}}\;{\text{images}} - {\text{Raw}}\;{\text{images}})$
Maean difference $\overline{{{\text{Diff}}}}$	$\overline{{{\text{Diff}}}} {\text{ = }}\frac{{\sum _{{{\text{i = 1}}}}^{n} {\text{Diff}}_{{\text{i}}} }}{{\text{n}}}$
Varaiance of difference $\overline{{{\text{Var}}}}$	$\overline{{{\text{Var}}}} = \frac{{\sum _{{i = 1}}^{n} ({\text{Diff}}_{i} - \overline{{{\text{Diff}}}} )^{2} }}{{n - 1}}$
Standard deviation $\:\text{SD}$	${\text{SD = }}\sqrt {\overline{{{\text{Var}}}} }$
T-value	$$\begin{gathered} {\text{t}}\;{\text{ = }}\frac{{\overline{{{\text{Diff}}}} }}{{\frac{{{\text{SD}}}}{{\sqrt {\text{n}} }}}} \hfill \\ \overline{{{\text{Var}}}} \hfill \\ \end{gathered}$$
Confidence interval $\:\text{CI}$	${\text{CI}} = \overline{{{\text{Diff}}}} \pm t \cdot \frac{{{\text{SD}}}}{{\sqrt n }}$

The initial step of the statistical paired t-test starts by organizing the data. Then calculate the basic difference between the accuracy of FCE images with raw images. Table 16 shows the performance of statistical paired t-test of FCAU-Net compared with the baseline models.

**Table 16:** Performance analysis of statistical significance testing with FCE images.

Model	Raw accuracy (%)	FCE accuracy (%)	$\overline{{{\text{Diff}}}}$	$\overline{{{\text{Var}}}}$	SD	t-value	DOF	*p*-value	95% CI (%)
DenseNet	68.27	69.43	1.16	0.16	0.40	2.90	4	0.045	0.03–2.29
VGG	71.23	72.62	1.39	0.16	0.40	3.47	4	0.025	0.31–2.47
AlexNet	72.91	73.51	0.60	0.16	0.40	1.50	4	0.20	−0.44–1.64
ResNet	76.25	77.44	1.19	0.16	0.40	2.98	4	0.042	0.12–2.26
U-Net	78.64	79.32	0.68	0.16	0.40	1.70	4	0.16	−0.30–1.66
Attention U-Net	82.36	83.78	1.42	0.16	0.40	3.55	4	0.023	0.36–2.48
FCAU-Net	90.51	99.89	9.38	0.25	0.50	42.00	4	< 0.001	8.50–10.26

The analysis shows that FCAU-Net achieved a mean improvement of 9.38%, with low variance across repeated runs. The resulting t-value of 42.0 and a *p*-value < 0.001 confirm that the improvement is highly statistically significant. The 95% confidence interval [8.50%, 10.26%] further reinforces that FCAU-Net consistently outperforms other models when leveraging FCE ultrasound images.

## Computational complexity analysis of FCAU-Net

In addition to superior accuracy in PCOS detection, the computational efficiency of the proposed FCAU-Net is a critical factor in evaluating its practical applicability of the model. In order to comprehensively evaluate the computational efficiency of the proposed FCAU-Net, several key metrics were considered across both raw and FCE ultrasound images. Training metrics included the training time per epoch, convergence rate, and CPU utilization during training. These metrics provide insights into how quickly the network learns from data, how efficiently it uses hardware resources, and how long it takes to reach optimal performance. Inference metrics encompassed inference time per image, inference time per batch, throughput with the number of images processed per second, and latency. These inference metrics measure the speed and responsiveness of the model. The training and inference metrics performance comparison is shown in Tables 17 and 18 respectively. The inference performance of the proposed FCAU-Net demonstrates significant improvements over conventional CNN models in terms of speed, throughput, and latency, highlighting its suitability for real-time PCOS diagnosis. On raw images, FCAU-Net achieves an inference time of 9.1 ms per image, which is considerably faster than other CNN models (Table 18). This reduced processing time allows for higher throughput, with FCAU-Net processing approximately 11.1 images per second, outperforming all other models in practical efficiency.

**Table 17:** Training metrics of FCAU-Net model.

Model	Training time per epoch (minutes)		Convergence rate (epochs)		CPU utilization (%)	
	Raw images	FCE images	Raw images	FCE images	Raw images	FCE images
DenseNet	6.0	6.1	35	34	60	62
VGG	8.0	7.8	40	39	65	63
AlexNet	5.0	4.9	30	29	55	56
ResNet	7.0	6.8	32	31	58	59
U-Net	8.5	8.3	38	37	60	61
Attention U-Net	9.0	8.8	40	39	62	63
Proposed FCAU-Net	6.8	6.5	28	27	54	55

Similarly, for FCE-processed images, FCAU-Net maintains a rapid inference time (Table 18) of 8.7 ms per image, which is again lower than the other CNN, resulting in a throughput of 11.5 images per second. The superior inference performance is attributed to the optimized architecture of FCAU-Net, which combines lightweight attention modules with feature-calibrated fusion, reducing redundant computations while focusing on the most informative regions of the ultrasound images.

**Table 18:** Inference metrics of FCAU-Net model.

Model	Inferenc time per image (ms)		Inferenc time per batch (ms)		Throughput (images/sec)		Latency (ms)	
	Raw images	FCE images	Raw images	FCE images	Raw images	FCE images	Raw images	FCE images
DenseNet	12.5	12.1	120	115	8.0	8.3	12.5	12.1
VGG	14.2	13.8	135	130	7.4	7.7	14.2	13.8
AlexNet	10.4	10.1	100	95	9.6	9.9	10.4	10.1
ResNet	11.8	11.4	110	108	9.1	9.3	11.8	11.4
U-Net	15.6	15.0	150	145	6.7	7.0	15.6	15.0
Attention U-Net	16.3	16.0	160	155	6.3	6.5	16.3	16.0
Proposed FCAU-Net	9.1	8.7	90	85	11.1	11.5	9.1	8.7

To evaluate model complexity and computational load, the analysis included the total number of learnable parameters, floating-point operations (FLOPs), and computational overhead introduced by network components such as attention modules and feature-calibrated fusion layers. Table 19 shows the analysis of model complexity and efficiency metrics that highlights the computational advantages of the proposed FCAU-Net over conventional CNN models for both raw and FCE ultrasound images. In terms of number of parameters, FCAU-Net maintains a relatively compact size of 7.3 million, which is lower than other CNN, reflecting its efficient design without compromising representational power. Regarding computational operations, FCAU-Net achieves a low FLOPs count for FCE images, which is significantly lower than other CNN, indicating that it requires fewer floating-point operations to generate predictions. This reduced computational load directly translates into faster processing and lower energy consumption. Furthermore, the computational overhead of FCAU-Net is categorized as low, in contrast to the high overhead observed in other CNN models, due to the optimized integration of attention mechanisms and feature-calibrated fusion modules that selectively process the most informative regions of the images. Overall, these metrics demonstrate that FCAU-Net achieves a balanced trade-off between high accuracy and computational efficiency, making it well-suited for automated PCOS detection.

**Table 19:** FCAU-Net model complexity and efficiency metrics.

Model	Number of parameters (M)		FLOPS ( X 10^9^)		Computation overhead	
	Raw images	FCE images	Raw images	FCE images	Raw images	FCE images
DenseNet	8.1	8.1	5.6	5.5	Moderate	Moderate
VGG	14.7	14.7	7.8	7.7	High	High
AlexNet	5.6	5.6	3.5	3.4	Low	Low
ResNet	11.2	11.2	6.7	6.6	Moderate	Moderate
U-Net	12.8	12.8	9.4	9.3	High	High
Attention U-Net	14.2	14.2	10.2	10.1	High	High
Proposed FCAU-Net	7.3	7.3	4.9	4.8	Low	Low

## Performance comparison with State-of-the-art models

To evaluate the effectiveness of the proposed FCAU-Net, a detailed comparison is done with existing state-of-the-art (SOTA) approaches for PCOS detection with accuracy metrics. Table 20 summarizes the performance of several recent techniques, including conventional CNNs, hybrid networks, attention-based models, and ensemble methods compared with the proposed FCAU-Net.

**Table 20:** Performance analysis of SOTA methods.

Model	Accuracy (%)
AResUNet^1^	98.00
Enhanced U-Net + ResNet^3^	97.80
GAN + CNN^4^	96.00
PCOS-WaveConvNet^6^	97.90
PCONet^23^	98.12
VGGNet16 + Stacking Ensemble^8^	98.90
VGG16 (modified last 4 layers)^10^	92.11
ASPPNet + ResNet^11^	98.79
CNN + BiLSTM^12^	97.74
ESDPCOS (CNN + GLCM)^13^	96.06
AMCNN^14^	98.79
CNN + KNN clustering^15^	97.00
MLOD^16^	96.00
Ocys-Net^17^	95.93
HHO-DQN^18^	96.50
ITL-CNN^19^	98.90
Ensemble (VGG16, ResNet50, MobileNet)^20^	95.00
Watershed + contour analysis^21^	97.80
CR-UNet^22^	91.20
Hybrid CNN^23^	95.00
SqueezeNet^26^	97.63
InceptionV3 + TL^28^	98.48
2D CNN + SVM, DT, RF^29^	98.07
Sequential 2D CNN + wrapper FS^31^	98.67
Elman NN + Gabor Wavelet^32^	78.10
BPA (modified LM optimization)^35^	93.92
EfficientNetB6 + Attention UNet^38^	98.12
Threshold-based segmentation^39^	97.00
DLNNSVM^40^	97.32
GrabCut + FL-SNNM^41^	97.99
GIST-MDR^42^	93.82
QEI-SAM^63^	99.31
Deeplabv3^64^	94.60
CystNet^68^	97.82
TL-CNN^69^	97.20
DC-UNet^70^	97.54
AdaResU-Net^71^	98.47
FCAU-Net	99.89

The SOTA methods demonstrates extensive exploration of neural-based systems for PCOS detection from ultrasound images, largely involving U-Net variants, hybrid segmentation-classification designs, and feature fusion strategies. However, existing approaches such as AResU-Net^1^ and CystNet^68^ primarily focus on architectural modifications or preprocessing filters without deeply modeling contextual dependencies and multi-level feature interactions. The proposed CAU-Net uniquely addresses these deficits by integrating a FFC module that jointly encodes spatial and contextual cues, enhancing discriminative representation across ovarian regions. Unlike CystNet threshold-based segmentation or AResU-Net residual attention layers focused on feature refinement, the FFC module adaptively weighs local-global dependencies through contextual recalibration, achieving superior follicle delineation and classification precision. Moreover, adding FCE preprocessing distinguishes FCAU-Net from conventional CLAHE preprocessing schemes, improving cyst boundary clarity and model generalization. As summarized below, FCAU-Net’s 99.89% detection accuracy notably exceeds SOTA methods, underscoring its advancements in both architectural and preprocessing aspects.

The results of fold-5 cross-validation demonstrate that the proposed FCAU-Net outperforms a wide range of SOTA techniques in PCOS detection from ultrasound images. Conventional CNN-based architectures were surpassed by FCAU-Net by margins ranging from 1% to 3%, highlighting its superior ability to extract and utilize discriminative features from ovarian ultrasound images. Furthermore, models relying on classical feature extraction methods performed significantly lower, emphasizing the advantage of FCAU-Net with attention and feature calibration mechanisms. The proposed FCAU-Net integrates feature-calibrated attention modules that selectively focus on informative regions of the ultrasound images, which likely accounts for its superior performance. In addition, its robust architecture ensures consistent performance across different folds, demonstrating both high accuracy and reliability.

## Ablation study on FCAU-Net

To rigorously evaluate the effectiveness of the architectural components integrated into the proposed FCAU-Net, an extensive ablation study is done to analyze the performance. The purpose of this analysis is to isolate and quantify the contribution of each key component namely FFCM, the modified attention gate, and the skip connections towards the overall performance of the network in detecting PCOS from ultrasound images. While the baseline U-Net serves as the foundational reference, progressive modifications and module additions allow us to systematically examine how each enhancement improves the network’s learning capability and discriminative power. This ablation study was performed on raw ultrasound images and FCE images. The ablation framework involved testing eleven different model variants, ranging from a simple baseline U-Net to progressively enhanced versions with either FFCM, default attention gates, modified attention gates. Table 21 shows the ablation study performance analysis of the FCAU-Net for raw images. Table 22 shows the ablation study performance analysis of the FCAU-Net for FCE images. The ablation results on both raw and FCE enhanced ultrasound images highlight the incremental contributions of each architectural component in FCAU-Net.

**Table 21:** Ablation study performance with Raw images.

Model	With FCE images (%)			
	Accuracy	Precision	Recall	F1-score
Baseline U-Net	78.64	79.1	78.0	78.5
U-Net without FFCM	79.10	79.5	78.6	79.0
U-Net with FFCM	80.25	81.0	80.0	80.4
U-Net with Default Attention Gate	81.36	82.0	81.2	81.3
U-Net with Modified Attention Gate	82.10	82.6	81.9	82.2
FCAU-Net without FFCM and Attention Gate	83.02	83.6	82.8	83.1
FCAU-Net without FFCM and Default Attention Gate	85.12	85.8	84.7	85.2
FCAU-Net without FFCM and Modified Attention Gate	86.27	87.0	85.9	86.4
FCAU-Net with FFCM and without Default Attention Gate	88.15	88.7	87.6	88.1
FCAU-Net with FFCM and without Modified Attention Gate	89.10	89.6	88.8	89.2
Proposed FCAU-Net	90.51	91.2	90.0	90.6

Starting from the baseline U-Net (Table 22) providing modest accuracy, the addition of FFCM or attention gates individually improves performance by enabling more effective feature representation and contextual learning. The U-Net variants with default or modified attention gates perform better than those with FFCM alone, underscoring the importance of selective focus in ovarian structure segmentation.

**Table 22:** Ablation study performance with FCE images.

Model	With FCE images (%)			
	Accuracy	Precision	Recall	F1-score
Baseline U-Net	79.32	79.8	78.9	79.3
U-Net without FFCM	80.10	80.6	79.5	80.0
U-Net with FFCM	81.56	82.0	81.0	81.4
U-Net with Default Attention Gate	82.74	83.2	82.3	82.7
U-Net with Modified Attention Gate	83.56	84.0	83.2	83.6
FCAU-Net without FFCM and Attention Gate	84.60	85.1	84.0	84.5
FCAU-Net without FFCM and Default Attention Gate	86.78	87.4	86.3	86.8
FCAU-Net without FFCM and Modified Attention Gate	88.15	88.7	87.6	88.1
FCAU-Net with FFCM and without Default Attention Gate	96.24	96.7	95.8	96.2
FCAU-Net with FFCM and without Modified Attention Gate	97.12	97.6	96.8	97.2
Proposed FCAU-Net	99.89	99.9	99.8	99.9

The modified attention gate consistently outperforms the default gate, reflecting the benefit of refining the gating mechanisms for ultrasound images. The highest performance is obtained with the full FCAU-Net, achieving 90.51% on raw images and an impressive 99.89% on FCE images, demonstrating that integrating both FFCM and modified attention gates yields the most discriminative and robust feature learning.

## Conclusion and future enhancements

This study proposes the FCAU-Net model, an enhanced Attention U-Net integrated with a Feature Fusion Context Module (FFCM), for classifying PCOS-infected ultrasound images with high accuracy. The methodology introduces two significant contributions. First, the dataset was preprocessed using image cropping, focusing on the main contextual regions by identifying extreme points and contours, followed by enhancement through FCE imaging. These steps emphasize high-intensity pixel features, ensuring better input quality for classification. Second, the FFCM was integrated into the Attention U-Net to optimize feature maps by fusing positional and contextual information, enhancing both deep and shallow features. Before augmentation, the dataset was partitioned to ensure that only original images were used for testing with 360 images, while augmented samples were exclusively utilized for training to enhance model generalization and robustness. The refined pipeline included data augmentation, resulting in a dataset of 45,600 images, divided into 80:20 for training and validation. Comparative evaluation against models like DenseNet, VGG, AlexNet, UNet, and Attention U-Net demonstrated the superior performance of FCAU-Net, achieving a classification accuracy of 99.89%, significantly outperforming existing approaches.

While FCAU-Net exhibits remarkable performance, challenges remain in further optimizing the encoding and decoding blocks with alternative loss functions and advanced optimizers. Although FCAU-Net incorporates feature-calibrated attention modules to focus on informative regions, very small or overlapping follicles with subtle intensity differences can still pose challenges, leading to occasional misclassification or missed detections. The proposed FCAU-Net is highly depended on high-quality ultrasound images, so the segmentation accuracy and detection of follicle is affected for images with severe noise, motion artifacts, or poor contrast. Additionally, FCAU-Net primarily focuses on morphological features visible in 2D ultrasound images and may not fully leverage temporal or volumetric information available in 3D or cine ultrasound scans, which could provide richer diagnostic cues. To overcome these limitations, future research could explore the integration of self-supervised or semi-supervised learning strategies that may enhance feature robustness. Additionally, hybrid architectures combining FCAU-Net with lightweight transformer modules or adaptive post-processing techniques could further improve the detection of subtle and overlapping follicles. The future work could focus on robust pre-processing and denoising techniques to enhance performance on low-quality or noisy images. Integrating 3D ultrasound data or temporal sequences into FCAU-Net could capture additional structural and dynamic information, potentially improving detection of small or overlapping follicles. Furthermore, incorporating explainable AI techniques such as attention heatmaps or feature attribution maps can enhance model interpretability. The proposed FCAU-Net may also focus on extending the FFCM with additional position and context blocks to further refine feature map optimization. By addressing these limitations, future iterations of FCAU-Net could achieve even higher reliability, generalizability, and practical usability for automated PCOS diagnosis.

## Data Availability

The dataset used in and associated metadata used for model development and evaluation can be accessed and this study is publicly available on Kaggle at the following link: https://www.kaggle.com/datasets/anaghachoudhari/pcos-detection-using-ultrasound-images All ultrasound images downloaded from this repository under the terms and conditions specified by the dataset provider
